# Verteporfin is a substrate-selective γ-secretase inhibitor that binds the amyloid precursor protein transmembrane domain

**DOI:** 10.1016/j.jbc.2022.101792

**Published:** 2022-03-03

**Authors:** Manuel A. Castro, Kristine F. Parson, Ilyas Beg, Mason C. Wilkinson, Kamila Nurmakova, Iliana Levesque, Markus W. Voehler, Michael S. Wolfe, Brandon T. Ruotolo, Charles R. Sanders

**Affiliations:** 1 Department of Biochemistry, Vanderbilt University, Nashville, Tennessee, USA; 2 Center for Structural Biology, Vanderbilt University, Nashville, Tennessee, USA; 3 Department of Chemistry, University of Michigan, Ann Arbor, Michigan, USA; 4 Department of Medicinal Chemistry, University of Kansas, Lawrence, Kansas, USA; 5 Chemical and Physical Biology Program and Center for Structural Biology, Vanderbilt University, Nashville, Tennessee, USA

**Keywords:** amyloid precursor protein, γ-secretase, screening, membrane, inhibitor, Aβ, amyloid-β polypeptide, APP, amyloid precursor protein, C99, C-terminal 99 amino acid fragment of APP, DDMB, β-n-dodecyl melibioside, IM-MS, ion mobility–mass spectrometry, LMPG, 1-myristoyl-2-hydroxy-sn-glycero-3-phospho-(1′-rac-glycerol)

## Abstract

This work reports substrate-selective inhibition of a protease with broad substrate specificity based on direct binding of a small-molecule inhibitor to the substrate. The target for these studies was γ-secretase protease, which cleaves dozens of different single-span membrane protein substrates, including both the C99 domain of the human amyloid precursor protein and the Notch receptor. Substrate-specific inhibition of C99 cleavage is desirable to reduce production of the amyloid-β polypeptide without inhibiting Notch cleavage, a major source of toxicity associated with broad specificity γ-secretase inhibitors. In order to identify a C99-selective inhibitors of the human γ-secretase, we conducted an NMR-based screen of FDA-approved drugs against C99 in model membranes. From this screen, we identified the small-molecule verteporfin with these properties. We observed that verteporfin formed a direct 1:1 complex with C99, with a K_D_ of 15–47 μM (depending on the membrane mimetic used), and that it did not bind the transmembrane domain of the Notch-1 receptor. Biochemical assays showed that direct binding of verteporfin to C99 inhibits γ-secretase cleavage of C99 with IC_50_ values in the range of 15–164 μM, while Notch-1 cleavage was inhibited only at higher concentrations, and likely *via* a mechanism that does not involve binding to Notch-1. This work documents a robust NMR-based approach to discovery of small-molecule binders to single-span membrane proteins and confirmed that it is possible to inhibit γ-secretase in a substrate-specific manner.

The *APP* gene encodes the amyloid precursor protein (APP), a single-span membrane protein implicated in neurological development, axon guidance, learning and memory, and general neuronal homeostasis (1, 2, 3). Heritable mutations affecting the *APP* gene were the first to be discovered to cause Alzheimer’s disease (AD) (4, 5, 6). Gene duplication of *APP* as well as a number of missense mutations in *APP* found in and around the region encoding the amyloid-β (Aβ) domain are associated with inherited forms of AD (1, 7, 8, 9, 10). Historically, these associations between genetics and AD have been thought to be the consequences of toxic oligomer and amyloid fibril formation by Aβ polypeptides released after successive proteolysis of APP by the β- and γ-secretase proteases (Fig. 1*A*) (7, 11, 12). These proteolytic events and the subsequent aggregation of Aβ—referred to as “the amyloidogenic pathway”—have been extensively targeted pharmacologically (13, 14). However, previous clinical trials of inhibitors of γ-secretase have failed, in large part due to toxicity associated with lack of substrate-specific inhibition of γ-secretase, which has many dozens of substrates (15, 16). Particularly notable is toxicity resulting from inhibition of Notch-1 cleavage, which disrupts essential signaling from this receptor (17, 18). In this paper, we describe our efforts to find compounds that act as substrate-selective γ-secretase inhibitors. Specifically, we sought to discover compounds that inhibit cleavage of C99—the product of APP cleavage by β-secretase and the immediate precursor of Aβ, while at the same time allowing Notch cleavage by γ-secretase to proceed uninhibited.

**Figure 1:**
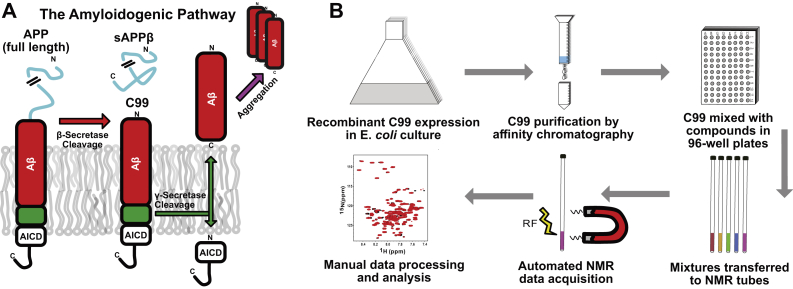
**The amyloidogenic pathway and our screening workflow for compounds that bind to the C99 protein**. *A*, Aβ amyloidogenesis begins with proteolysis of the full-length amyloid precursor protein (APP) by β-secretase, liberating the soluble extracellular domain (sAPPβ) and the transmembrane 99 residue C-terminal (C99) domain. C99 is then further proteolyzed by the γ-secretase complex in its transmembrane domain, generating both the amyloid intracellular domain (AICD) and Aβ, the latter of which can then go on to form Aβ oligomers and amyloid plaques. *B*, for high-throughput C99 screening, C99 is expressed in *E. coli* cultured in ^15^N isotopically enriched media and purified from cells by affinity chromatography into detergent micelle solutions suitable for solution NMR spectroscopy. The protein in NMR conditions is then mixed with small-molecule binding candidates (in DMSO) in 96-well plates and transferred to an NMR tube. NMR data are collected and then processed and analyzed. Standard NMR sample conditions are: 200 μl of 50 μM C99 in 2.5% w/v LMPG, 50 mM PIPES, 100 mM NaCl, 0.5 mM EDTA, pH 6.5, 318 K (45 °C), and 50 μM of the test compound, all in a 3 mm NMR tube.

In addition to their potential utility in reducing Aβ production, compounds that inhibit C99 cleavage would be valuable tool compounds for studying C99 biology. Intact C99 may be directly involved in some of the cellular pathologies associated with AD, such as defects in mitophagy, autophagy, mitochondrial structure, bioenergetics, lipid homeostasis, and AD-model animal behavior (19, 20, 21, 22, 23, 24, 25). These discoveries suggest that C99 is itself directly involved in AD pathogenesis. Thus, a C99-specific binder that selectively inhibits its degradation would be a useful tool for further investigating the relationship between C99 and AD.

We are not the first to consider the concept of substrate-selective modulators of amyloidogenesis (26, 27, 28, 29). Indeed, “Notch-sparing” modulators of γ-secretase that bind directly to the enzyme have been reported in the literature, with one entering clinical trials (30). This compound, however, possessed questionable selectivity for C99 (31) and caused toxicities due to suppression of Notch cleavage at higher doses (32). Moreover, some compounds initially reported to selectively modulate C99 cleavage by binding free C99 (26) were later shown NOT to bind free C99, but instead act by interacting directly with γ-secretase, perhaps cooperatively with C99-dependent binding (33, 34, 35, 36, 37). Another group reported the discovery of benzofuran compounds that appeared to bind full-length APP and inhibited its proteolysis by γ-secretase (29). Studies such as these motivated this study to further explore this substrate-directed inhibitory approach.

Here, we report the results of our search for compounds that act by specifically binding to the C99 substrate rather than by targeting the protease itself. We describe the discovery that the porphyrin-based drug verteporfin directly binds C99 in model membranes to form a 1:1 complex with a K_D_ of ca. 17 μM. Moreover, verteporfin inhibits γ-secretase cleavage of C99 at concentrations where it does not inhibit Notch cleavage.

## Results

### Optimization of conditions for screening for small molecules that bind C99

We previously determined the 3D structure of monodisperse C99 in model membranes using solution nuclear magnetic resonance (NMR) spectroscopy (PDB: 2LP1) (38). Previous studies from our lab established that NMR can be used to detect and characterize the interactions of C99 both with small-molecules such as cholesterol and with other proteins (33, 38, 39, 40, 41). We therefore set out to screen a library of 1184 FDA-approved drugs using NMR to detect C99-binders and then to exhaustively validate binding and to determine the binding affinity, as outlined in Figure 1.

**Figure 2:**
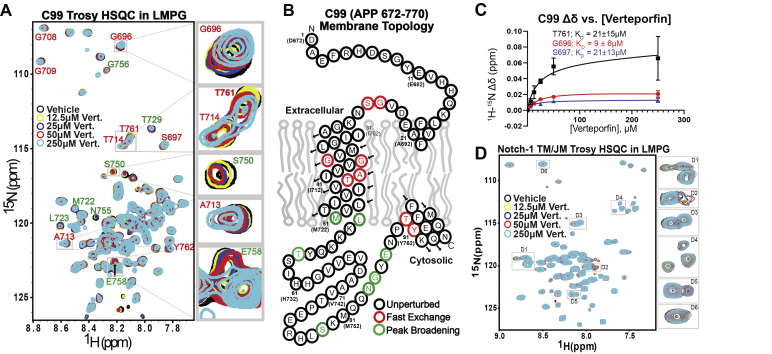
**Verteporfin binds C99 in LMPG micelles and does not bind to the Notch-1 TM/JM**. *A*, five overlaid C99 TROSY NMR spectra of C99 with varying concentrations of verteporfin. *B*, C99 topology diagram highlighting residues that undergo perturbations in response to verteporfin binding. Sample conditions: 50 μM C99 in 50 mM PIPES buffer, 100 mM NaCl, 0.5 mM EDTA, 2.5% w/v LMPG, pH 6.5, 318 K, 3 mm NMR tube. *C*, plotted chemical shift perturbations for the titration summarized in panel A. The average result of three replicate experiments was fit by a 1:1 binding model. Error bars indicate the standard error of the mean (SEM). *D*, TROSY spectral overlay for the Notch-1 TM/JM domain at varying concentrations of verteporfin. For more direct comparison with selected peaks in the C99 data (insets to panel A), peaks are highlighted in insets D1–D6, showing the minimal spectral perturbations seen for Notch in response to verteporfin treatment. The data shown in D have also been collected in three technical replicates. LMPG, 1-myristoyl-2-hydroxy-sn-glycero-3-phospho-(1′-rac-glycerol).

Before initiation of the screen, various NMR conditions such as the C99 protein concentration, detergent concentration, tolerable concentration of DMSO (vehicle), and NMR pulse programs/parameters were explored. While several conditions produced high-quality NMR spectra, we settled on screening conditions consisting of 50 μM C99 in 2.5% w/v (52 mM) lyso-myristoylphosphtidylglycerol (LMPG) micelles at pH 6.5, with NMR spectra being acquired using the BEST-TROSY pulse sequence (42, 43). These conditions were similar those used in our previous determination of the structure of C99 (38). Nearly all C99 resonances exhibited satisfactory signal-to-noise even at 50 μM protein concentration and using a short data acquisition time (35 min, Fig. 2*A*). This approach enabled the screening of all 1184 samples in 36 days with the assistance of a Bruker SampleJet automated sample changer.

### Verteporfin binds C99 avidly, but not Notch-1

Using the optimized conditions described above, we completed a screen of the available SelleckChemical library of FDA-approved drugs. We identified 20 compounds as potential hits based on the observation that 50 μM concentrations of these compounds perturbed the resonances of 50 μM C99 (Fig. S1). Of these 20 compounds, only verteporfin yielded results that stood out when C99 was subjected to a titration by each compound. Specifically, only verteporfin led to changes in the positions of C99 peaks during titration that were seen to saturate at submillimolar concentrations. Chemical shift perturbations were observed in TROSY amide backbone NH resonances from multiple C99 peaks, most arising from sites in and around the transmembrane domain and amphipathic C-helix (Fig. 2, *A* and *B*). For experiments in LMPG micelles, fits of the 1:1 binding model to the titration traces from multiple different peaks (Fig. 2*C*) led to determination of an average dissociation constant (K_D_) of 17 ± 11 μM across multiple residues in and around the TM domain. The presence of both fast and slow-exchanging resonances is consistent with the determined micromolar K_D_ value. Peaks that undergo large changes in resonance frequency upon complex formation fall in the slow exchange regime, where both complexed and free protein peaks are directly observed. Peaks that undergo smaller changes in frequency upon complex formation were in the fast exchange regime, where only a single peak is observed at a population-weighted average frequency between the free and fully complexed peak frequency limits.

We have previously used NMR to characterize the structure of the Notch-1 transmembrane domain flanked by its juxtamembrane segments (residues 1721–1771, “Notch-1 TM/JM”) (44). Titration of Notch-1 TM/JM by verteporfin in LMPG micelles under the same conditions as used for the titration of C99 revealed only minor perturbations of Notch resonances in response to increasing levels of verteporfin (Fig. 2*D*). That there are minor verteporfin-induced shifts that are small and much less extensively be seen than for C99 is not surprising in light of data shown below that verteporfin does bind nonspecifically to micelles. Overall, these NMR titration results indicate that verteporfin binds C99 but not Notch-1.

### Verteporfin binds to C99 in other membrane mimetics

**Figure 3:**
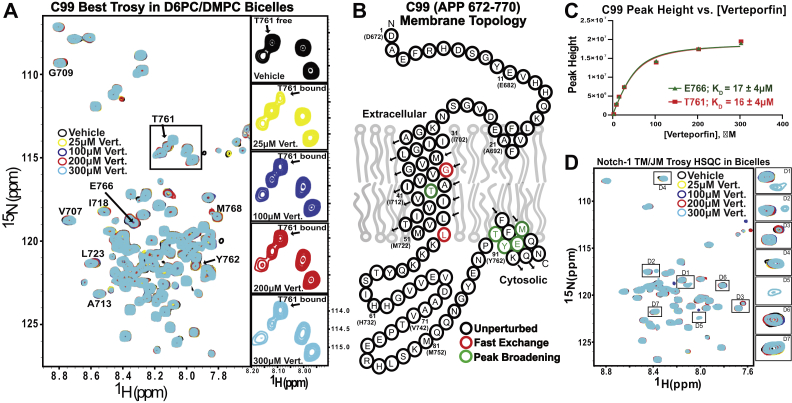
**Verteporfin binds C99 in D6PC/DMPC (q = 0.33) bicelles with an observed K**_**D**_**of 17 ± 4 μM, whereas verteporfin does not bind the Notch-1 TM/JM.***A*, five overlaid C99 TROSY NMR spectra of C99 at varying concentrations of verteporfin. The central insert zooms in on the boxed region near T761 and shows a new peak appearing in response to increasing verteporfin concentrations. The subpanels to the right illustrate the individual spectra of the T761 peak from the titration series. The free (0 M verteporfin) resonance of T761 disappears as the new bound state T761 resonance appears nearby in a verteporfin-dependent manner. *B*, topology plot for C99, highlighting sites for which verteporfin binding induced either gradual (fast exchange) changes in peak positions or the slow exchange disappearance of free protein peaks in concert with the appearance of the corresponding complexed-protein peaks. *C*, plotted chemical shift perturbations for two peaks from the titration shown in panel A. K_D_ values were derived from fitting these data to the one-site binding model, where error values were derived from the error of the fit. *D*, TROSY spectral overlay for the Notch-1 TM/JM at varying concentrations of verteporfin in bicelles, indicating that verteporfin does not bind to Notch-1. It is worth noting that spectral perturbations are noticeable at 300 μM verteporfin, which are shown as insets D1-D7. However, these weak effects are likely due to non-specific perturbation of the membrane mimetic. Data shown in this figure are n = 1, and the error shown in panel C are the errors of the fit.

To confirm that binding of verteporfin to C99 is not highly dependent on the nature of the membrane mimetic in which C99 is solubilized, we repeated TROSY-monitored titrations of C99 in two model membrane systems that differ in important ways from anionic LMPG micelles (1): *zwitterionic* bicelles composed of 3:1 (mol:mol, q = 0.33) dihexanoylphosphatidylcholine (D6PC):dimyristoylphosphotidylcholine (DMPC) and (2) *nonionic* beta-dodecylmelibiose (DDMB) micelles. NMR titration of C99 in D6PC-DMPC bicelles by verteporfin revealed both fast- and slow-exchange perturbations, similar to what was observed in LMPG (Fig. 3*A*). The perturbed residues (regardless of NMR exchange regime) mainly mapped to the transmembrane and amphipathic C-helix residues (Fig. 3*B*). Fitting of two of the titration curves revealed K_D_ of 17 ± 4 μM and K_D_ of 16 ± 4 μM, corroborating well with what was seen in LMPG micelles (Fig. 3*C*). A corresponding titration of the Notch-1 juxtamembrane/transmembrane domain in micelles by verteporfin again revealed only minor spectral changes at very high concentrations that are much less extensive than seen for C99 and likely arise from affects related to nonspecific binding of verteporfin to the protein-containing micelles and bicelles (Fig. 3*D*).

Verteporfin titration of C99 in nonionic DDMB micelles confirmed binding in this medium as well, with fitting of the data for two different peaks yielding K_D_ of 43 ± 17 μM and 47 ± 22 μM, slightly weaker than seen for LMPG micelles and DHPC-DMPC bicelles (Fig. S2, *A*–*C*). Again, the peaks that shifted were mostly located in the transmembrane domain and amphipathic C-helix. Verteporfin titration of Notch-1 in DDMB revealed little change in the spectrum of Notch-1 until a concentration of 300 μM (Fig. S2*D*), consistent with interactions between Verteporfin and Notch-1 that are only very weak and/or nonspecific.

The DHPC-DMPC bicelle and DDMB micelle results corroborate the LMPG micelle results to show that verteporfin binds C99 in a wide range of membrane-like conditions and that the affinity exhibits only modest variation (K_D_ in the range of 15–47 μM) across multiple different classes of model membranes.

### Verteporfin associates with membrane mimetics

**Figure 4:**
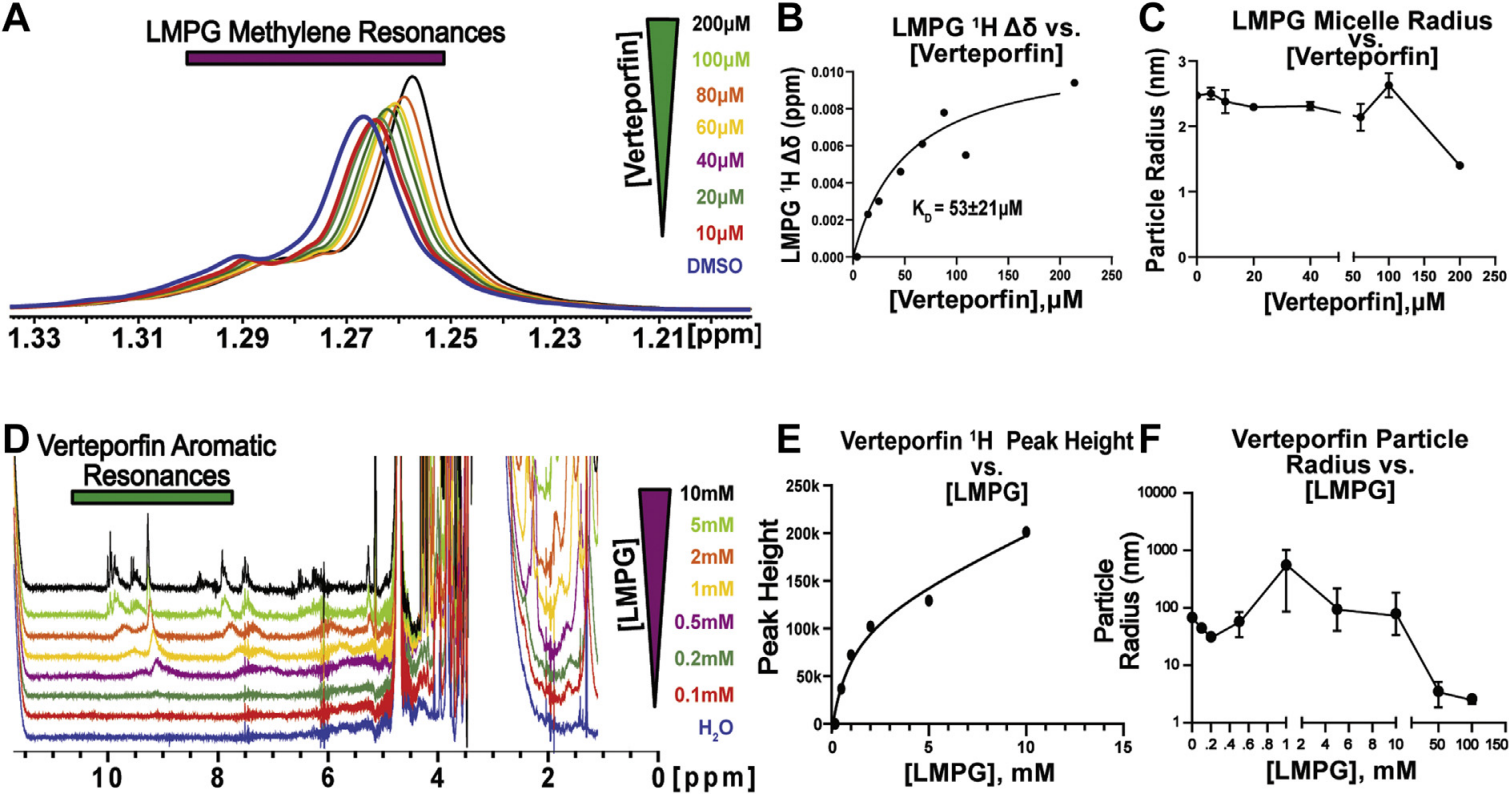
**Verteporfin binds to LMPG micelles, which suppress its aggregation in aqueous solution.***A*, overlaid 1D ^1^H NMR spectra showing the LMPG methylene resonances from empty LMPG micelles as verteporfin is titrated in. Sample conditions: 200 μl of 2.5% LMPG in 50 mM PIPES buffer, 100 mM NaCl, 0.5 mM EDTA, pH 6.5, 318 K, 3 mm NMR tube. *B*, LMPG micelle acyl chain methylene peak resonance chemical shift perturbations reveal binding of verteporfin to LMPG micelles. Data is n = 1. *C*, DLS measurements show that the size of the LMPG micelles under the same conditions as A is insensitive to verteporfin concentrations in the 0–200 μM range. Data is n = 3. *D*, overlaid 1D ^1^H NMR spectra of constant 100 μM verteporfin in NMR buffer with increasing concentrations of LMPG. 100 μM verteporfin in 50 mM PIPES buffer, 100 mM NaCl, 0.5 mM EDTA, pH 6.5, 318 K, 3 mm NMR tube. *E*, the peak heights of verteporfin aromatic resonances are increased (due to line-narrowing) in response to increasing LMPG concentration. Data is n = 1. *F*, DLS reveals that as 100 μM verteporfin is titrated with LMPG that soluble aggregates of verteporfin are fully dispersed by the point where the LMPG concentration reaches 50 mM. Data represents n = 3 technical replicates. For panels C and F, error bars reflect the SEM of triplicate experiments.LMPG, 1-myristoyl-2-hydroxy-sn-glycero-3-phospho-(1′-rac-glycerol).

We tested to see if verteporfin interacts with LMPG micelles in the absence of protein to investigate the possibility that verteporfin interactions with C99 might be modulated by the intrinsic affinity of verteporfin for membranes. We therefore titrated empty LMPG micelles with verteporfin, as monitored by examining the 1-D 1H NMR spectra of LMPG as a function of verteporfin concentration. Concentration-dependent changes in the position of the LMPG acyl chain methylene peak envelope revealed that their resonance frequencies were shifted by increasing verteporfin concentrations (Fig. 4*A*). A fit of the one verteporfin binding to one detergent micelle binding model to these changes yielded a reasonably good fit and a K_D_ of 53 ± 21 μM (Fig. 4*B*). Dynamic light scattering (DLS) measurements revealed that verteporfin had only modest effects on micelle particle size over a range of concentrations from 0 to 200 μM (Fig. 4*C*).

We also carried out a reciprocal titration in which the 1H NMR resonances from constant 100 μM verteporfin resonances were monitored as LMPG was titrated up through and far beyond its critical micelle concentration (CMC), which is ca. 0.2 to 0.5 mM at neutral pH values (45). Verteporfin resonances were very broad at LMPG concentrations below CMC (Fig. 4*D*), indicating aggregate formation by verteporfin in micelle-free aqueous solutions. Above CMC the quality of its spectrum increased gradually as the LMPG concentration was increased to 10 mM, suggesting dispersal of the constant population of verteporfin from interacting with a small number of micelles at LMPG concentrations near the CMC to interactions at higher LMPG levels in which each micelle has fewer associated verteporfin molecules (Fig. 4*E*). DLS measurements further illuminate that even in the mixture of 10 mM LMPG and 100 μM verteporfin, there is a persistence of large verteporfin aggregates, which are fully dissolved only near the point at which the LMPG concentration approaches that present in NMR screening conditions (50 mM) (Fig. 4*F*). This indicates that micelle formation is required to disperse the verteporfin aggregates and that the concentration of the micelles needs to be high enough to ensure that multiple molecules of verteporfin do not interact with same micelle.

Taken together, our results indicate that verteporfin binds to LMPG micelles, which suppresses verteporfin aggregation at higher micelle concentrations. The results also indicate that verteporfin does not significantly alter the size or structure of the micelles with which it associates, provided that there are a sufficient number of micelles present. We conclude that verteporfin’s membrane affinity helps bring this compound in contact with C99 by solubilizing the drug and by providing the model membrane environment in which these two molecules can encounter each other. However, it is notable that under identical conditions, Notch-1 in LMPG micelles does not exhibit interactions with verteporfin, indicating that micelle binding by verteporfin alone is not the determinant of C99–verteporfin interaction.

### 1H,1H-NOESY confirms direct contact between verteporfin and C99

To verify that that complex formation between verteporfin and C99 truly involves direct intermolecular contact, we recorded a 2D 1H,1H-NOESY spectrum for a 1:1 mixture of verteporfin and C99 in 2.5% d_27_-LMPG (deuterated acyl chains) micelles to see if cross-peaks can be observed between well-resolved verteporfin and C99 resonances. Fig. S3 documents the unambiguous presence of at least two such cross-peaks, which confirms direct binding of verteporfin to C99. Other verteporfin-C99 cross-peaks were also likely present but are difficult to unambiguously assign due to peak overlap with the intense resonances from the nondeuterated glycerol backbone of d_27_-LMPG. These data confirmed direct verteporfin–C99 interactions.

### Mutations in C99 reduce the affinity of verteporfin for C99

We tested to see if verteporfin binding to C99 could be attenuated or eliminated by mutations in the protein. The single-site mutations L723A, and T761A were chosen both because we observed chemical shift perturbations for these residues in one or all the previously discussed model membrane conditions and because of their dispersal across the C99 primary sequence. As shown in Fig. S4, we observed that binding was disrupted by both of these mutations and that peak shifts were either nonexistent or weak as a function of verteporfin concentration and could not be fit by binding isotherms, indicating that these residues played critical roles in mediating interactions between C99 and verteporfin. It is worth noting that peaks from transmembrane residues such as G708, G709, and A713 continued to undergo small shifts or broadening in the spectra from these mutants, likely reflecting the residual effects of verteporfin’s affinity for detergent micelles and forced cohabitation of verteporfin with C99 molecules at high verteporfin concentrations, as was also seen for Notch-1, but only at high verteporfin concentrations.

### Native ion mobility–mass spectrometry and chemical cross-linking indicate verteporfin binds to the monomeric form of C99 and does not induce homodimerization

We sought to determine whether verteporfin binds to the monomeric form of C99 and if binding of verteporfin induces a change in the oligomeric state of C99. C99 is known to form monomers or dimers depending on concentration, lipid composition, and model membrane type, or mutation (46, 47, 48). Glutaraldehyde (GA) cross-linking of verteporfin–C99 mixtures in LMPG micelles in NMR-like conditions showed no increase in higher-order C99 species upon increasing GA concentration when compared with the DMSO-only treated control (Fig. S5). This finding was confirmed by showing no change in oligomeric state when the GA concentration was maintained constant and verteporfin was increased. These results indicated both that verteporfin binds to monomeric C99 and that binding of verteporfin does not induce protein dimerization.

**Figure 5:**
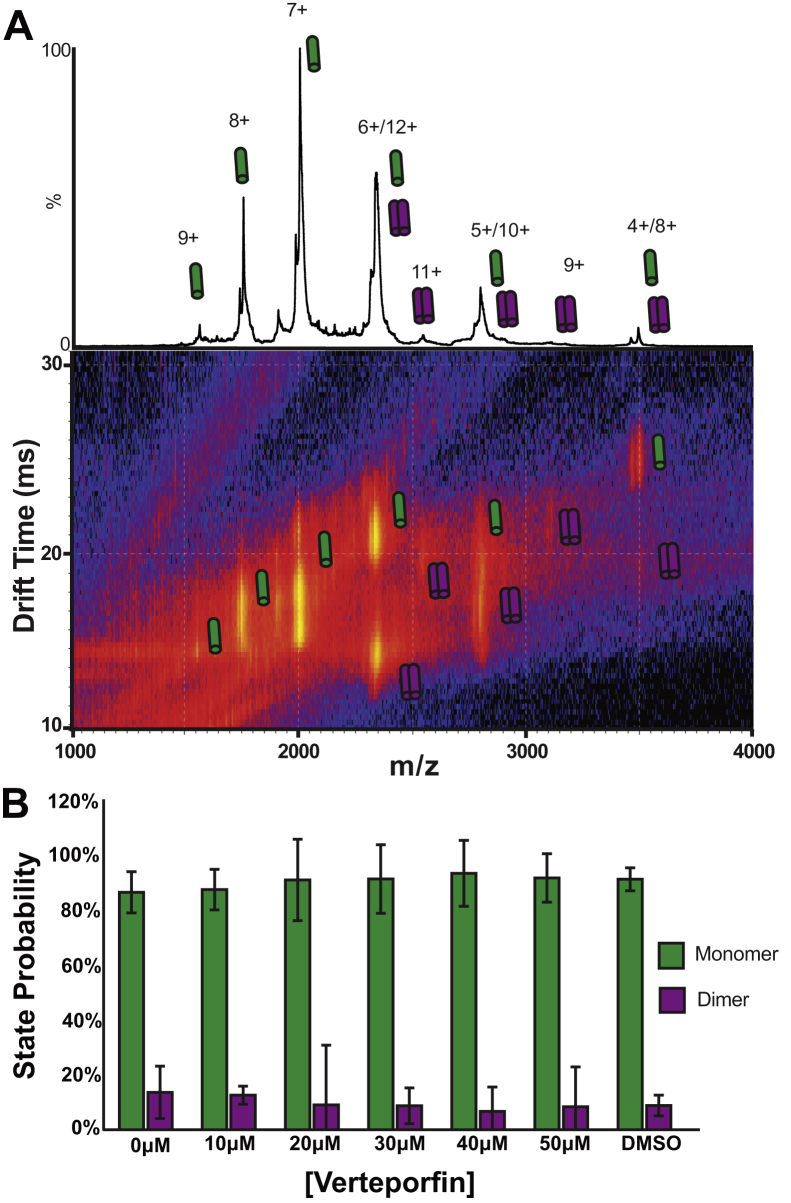
**IM-MS indicates that verteporfin does not alter C99 mass or induce dimerization.***A*, a representative mass spectrum (*top*) and IM-MS spectrum (*bottom*) showing the various states of C99 oligomerization. Native C99 monomer charge states (4–9+) are denoted by a single *green* cylinder, and dimer charge states (8–12+) are shown as two *purple* cylinders. No changes in C99 mass were observed, suggesting that no chemical modifications were made to the protein in response to drug treatment. *B*, the relative intensities of each species were used to calculate the percent of each oligomeric species for C99 at 0 μM, 10 μM, 20 μM, 30 μM, 40 μM, 50 μM verteporfin, respectively. To avoid overlapping monomer/dimer peaks, monomer charge states 9–11+ and dimer charge states 9+ and 11+ were included in analysis. Quantification of the IM-MS data indicates that C99 does not undergo any significant change in oligomeric state upon addition of verteporfin, indicating that verteporfin interacts with the C99 monomer. Data is n = 3, where error bars indicate the standard deviation of three technical replicate experiments. IM-MS, ion mobility–mass spectrometry.

Native ion mobility–mass spectrometry (IM-MS) has proven to be a useful structural biology tool capable of interrogating complex mixtures. With the use of membrane mimetics, the native conformation of membrane proteins can be retained and studied using IM-MS (49, 50, 51, 52, 53, 54). IM separates the ions based on their size, shape, and charge. In native IM-MS experiments, we can separate different conformations and oligomeric states of membrane protein ions (55, 56, 57, 58, 59). All IM-MS data were collected using a Synapt G2 HDMS IM-Q-ToF mass spectrometer (Waters), with a direct infusion nESI source set to positive ion mode. Instrument settings were tuned to dissociate detergent micelles, with minimal perturbation of protein structure prior to the IM separator. Arrival time distributions of charge states that were known to be uniquely monomer or dimer, as indicated in Figure 5, were extracted using a text-based format using TWIMExtract (60). IM-MS of C99–verteporfin mixtures in LMPG and DDMB micelles revealed that verteporfin causes only a marginal increase in the population of dimeric C99 in low detergent conditions (high protein-to-detergent ratio), which indicates verteporfin-induced dimerization would be insignificant at the much higher detergent concentrations (and lower C99-to-detergent ratios) of our NMR-based binding experiments (Fig. 5, *A*–*C*). We note that verteporfin was not detected in complex with C99 by IM-MS experiments, and this may be due to the dissociation of verteporfin with the surrounding detergent molecules during collision-induced dissociation.

The IM-MS data also revealed that the mass of C99 remained intact following complex formation with verteporfin, indicating no covalent modification of the protein by verteporfin or induced oxidation (Fig. 5*A*). This is a significant observation as verteporfin is a photoactivatable molecule that can stimulate the production of radical oxygen species (ROS) in the presence of ambient light and O_2_ (61). Stringent precautions were taken throughout these studies to ensure that verteporfin-containing samples were maintained in the dark. The IM-MS data confirmed that our observations are unrelated to potential photoactivation of verteporfin.

### Verteporfin inhibits γ-secretase cleavage of C99 with an approximate twofold selectivity over Notch-1

**Figure 6:**
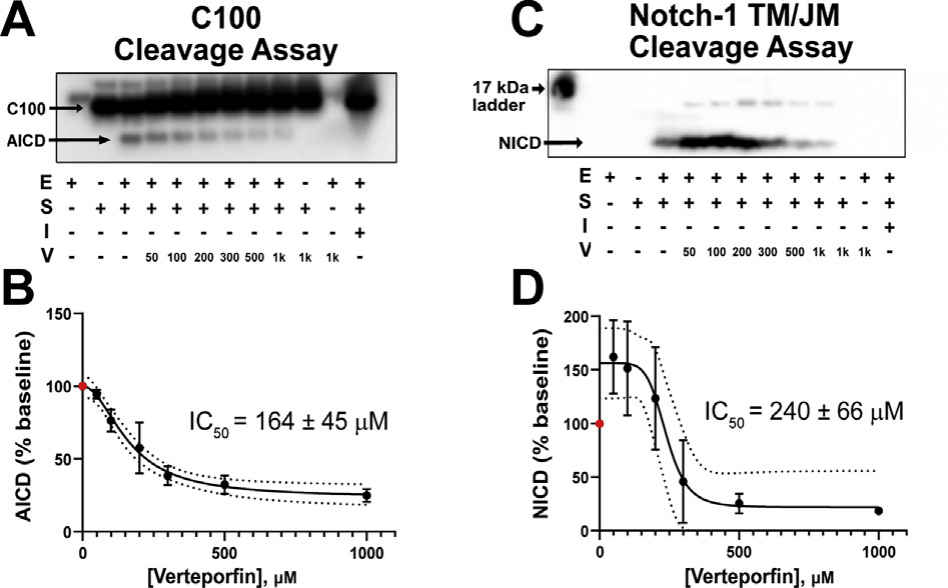
**Verteporfin inhibits γ-secretase cleavage of C100-Flag more potently than that for Notch-1.***A*, Anti-Flag tag Western blot of verteporfin inhibition of C100-Flag cleavage by 30 nM purified γ-secretase at different levels of verteporfin. The legend below the blot denotes the added components: **E is enzyme** (γ-secretase), **S is substrate** (C100), **I is inhibitor** (30 nM LY411,575), and **V is verteporfin** in micromolar units. Top band: C100-Flag substrate; bottom band; AICD-Flag (intracellular domain product of γ-secretase cleavage of C100-Flag). The presence of both enzyme and substrate produces the amyloid intracellular domain (AICD) band, indicating C100 cleavage. Upon addition of increasing amounts of verteporfin, the cleavage efficiency is greatly reduced in a concentration-dependent manner. The AICD band intensities were quantified using densitometry using ImageJ, as shown in the next panel (3 replicates). *B*, quantitation of the data for verteporfin inhibition of AICD production from panel A (plus replicates). “% Baseline” refers to the amount of AICD that is observed relative to the 0 verteporfin conditions. These data indicate an IC_50_ of 164 ± 45 μM (n = 3, technical replicates). *C*, Western blot of the γ-secretase assay showing cleavage of Notch-1 TM/JM to release NICD, as detected using an antibody against the V1744 neoepitope. Notch-1 TM/JM is recombinant Notch-1 transmembrane/juxtamembrane construct (residues 1721–1771). The legend below the blot denotes the added components: **E is enzyme** (γ-secretase), **S is substrate** (Notch-1 TM/JM), **I is inhibitor** (30 nM LY411,575), and **V is verteporfin** in micromolar units. The presence of both enzyme and substrate produces the Notch Intracellular Domain (NICD) band, indicating Notch cleavage. It is seen in these data that verteporfin first activates then inhibits γ-secretase cleavage of Notch-1. The NICD band intensities were quantified using densitometry (plus results for two additional replicates) using ImageJ, as shown in the next panel. *D*, quantitation of the (n = 3) data for verteporfin inhibition of NICD production from panel C. ”% baseline” refers to the amount of NICD that is observed relative to the 0 verteporfin conditions. The error for the dataset was propagated using the standard deviation of three technical replicates. Upon addition of increasing amounts of verteporfin, it is seen that Notch-1 cleavage is bi-phasic: cleavage is first activated by verteporfin until a concentration of 200 μM, then inhibited at higher concentrations, with an estimated IC_50_ of 240 ± 66 μM (n = 3). For both the C100 and Notch cleavage data presented here, uncropped blots are shown in the Supporting Information (Fig. S7), with molecular weight markers for the Notch cleavage reaction being presented there.

We tested whether formation of the C99–verteporfin complex resulted in inhibition of C99 cleavage by γ-secretase. γ-Secretase assays were conducted *in vitro* for both C100 (C99 with an N-terminal start methionine) and Notch-1 TM/JM as substrates (Fig. 6, *A*–*D*, respectively). Verteporfin induced dose-dependent inhibition of γ-secretase-catalyzed production of the amyloid intracellular domain (AICD), with an IC_50_ of 164 ± 45 μM under these assay conditions. (Fig. 6*B*) The degree of inhibition levels off at approximately 75%, with some residual cleavage activity being observed even at apparently saturating levels of verteporfin. In another condition, where the enzyme concentration was much lower, verteporfin exhibited a 15 ± 1 μM IC_50_ for γ-secretase cleavage of C99 (Fig. S6) which correlated well with the observed K_D_ values between verteporfin and C99 (Figs. 2, 3 and S2). The lower IC_50_ observed in this experiment relative to that of Figure 6, *A* and *B* reflects the lower concentration of γ-secretase: With less enzyme in competition for C99 binding, verteporfin can more effectively protect C99 from proteolytic processing.

For Notch-1 (Notch-1 TM/JM), concentrations of verteporfin below 200 μM actually *enhanced* Notch-1 cleavage by γ-secretase relative to 0 verteporfin conditions (Fig. 6, *C* and *D*). However, at higher concentrations, verteporfin inhibited γ-secretase cleavage of Notch-1 with an IC_50_ of 240 ± 66 μM. As for C99, saturating concentrations of verteporfin reduced cleavage by approximately 75%.

Taken together, these data indicate that at lower concentrations verteporfin binding to C99 shields the protein from γ-secretase cleavage without inhibiting γ-secretase cleavage of Notch. For example, when the concentration of verteporfin is at the IC_50_ of 164 μM, there is 50% maximal inhibition of C99 cleavage, but no inhibition of Notch-1 cleavage (Fig. 6). Moreover, the fact that verteporfin was not seen by NMR to bind to Notch under three different sets of model membrane conditions, but can nevertheless inhibit Notch cleavage at higher concentrations, suggests that inhibition is likely be the consequence of interactions of high levels of verteporfin with γ-secretase and/or with the model membranes employed in the γ-secretase assays.

### Modeling the C99–verteporfin complex

**Figure 7:**
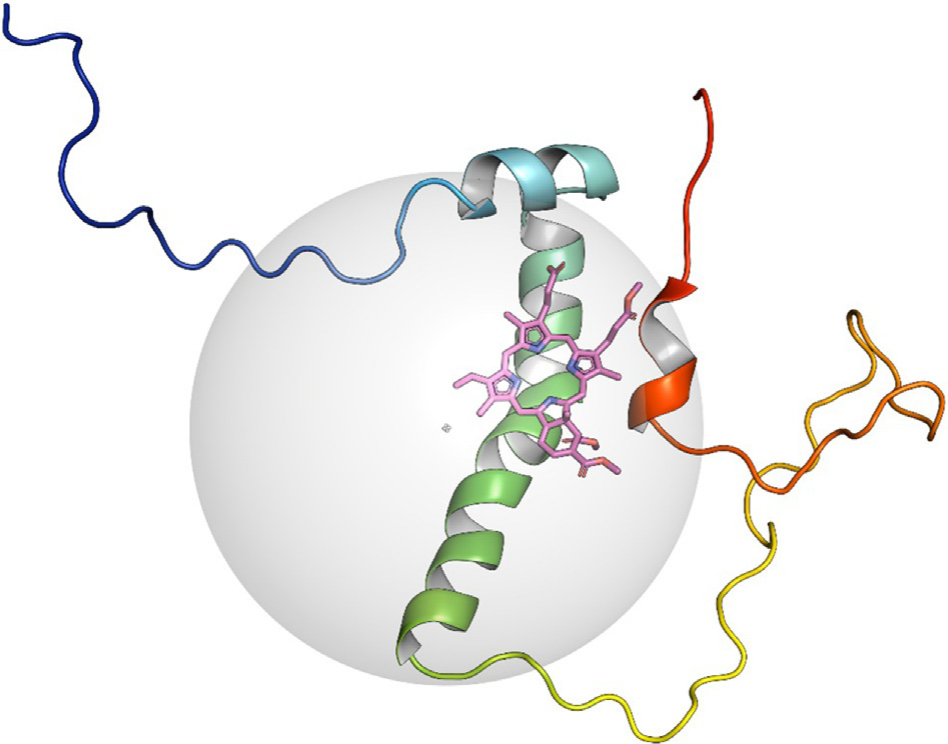
**Most favorably-scoring model from experimentally restrained Rosetta modeling of the micellar verteporfin-C99 complex**. Experimentally restrained Rosetta suggests that the planar face of verteporfin interacts with the transmembrane domain. It appears the entire macrocycle of the compound is embedded in the micellar model membrane and contacts transmembrane residues near the transmembrane kink. The positions of the semipolar methyl ester chains generally point towards the solvent-membrane interface and form contacts with the N- and C-terminal amphipathic helices. The gray sphere represents a detergent micelle.

Although verteporfin induced spectral perturbations across many regions of C99, the focal points were primarily in and around the transmembrane domain and amphipathic C-helix. We sought to use experimentally restrained Rosetta modeling to generate structural insight on how the complex may look and to illuminate possible mechanisms of γ-secretase inhibition. Using our previously published C99 structure (PDB: 2LP1) as a template, we generated models of the complex showing that verteporfin localizes to the transmembrane domain (Fig. 7). In the top-scoring model, verteporfin is seen to adopt a binding pose in which its planar macrocyclic ring is in contact with transmembrane C99 residues near the TM kink. The semipolar verteporfin methyl ester arms extend toward the surface of the membrane and may be responsible for the chemical shift perturbations seen in the amphipathic N- and C-helices.

## Discussion

APP can be classified as a member of the single-pass transmembrane receptors (SPTMRs), a collection of membrane proteins that make up approximately 6% of the human protein-coding genome and play critical roles in essential biological processes such as cell growth, adhesion, metabolism, and immunity (62). The close relationship between SPTMRs and human health and disease suggests that many proteins of this class may be viable drug targets; however, their transmembrane domains have been thought to lack the druggable pockets that are associated with multispan membrane or water-soluble proteins (63). Nevertheless, at least one drug does indeed target the transmembrane domain of an SPTMR. Eltrombopag is an FDA-approved thrombopoietin receptor (TPoR) agonist that activates the receptor by binding to its transmembrane domain (64, 65, 66). The present study was inspired, in part, by the hope that the paradigm in molecular recognition established by the Eltrombopag/TPoR interaction could be extended to the discovery of molecules that specifically bind to the transmembrane C99 domain of the human APP.

Our study establishes that verteporfin binds specifically to monomeric C99 with an affinity in the 10 to 50 μM range under a variety of model membrane conditions. Verteporfin does not induce dimerization of C99. Future work will be required to determine if a medicinal chemistry campaign can identify verteporfin analogs or derivatives with an even higher affinity for C99. Binding of verteporfin to C99 is facilitated by the intrinsic affinity of this compound for membranes, which increases the local concentration of this compound in the vicinity C99. NMR NOE measurements confirmed that the C99–verteporfin interaction is direct. Moreover, the fact that NOEs were seen between verteporfin and *both* C99 and detergent under conditions in which the vast majority of verteporfin was C99-associated strongly suggests that the C99–verteporfin interactions are at the detergent–protein interface. The fact that verteporfin binds C99 with similar affinity in LMPG micelles (anionic), dodecylmelibioside micelles (nonionic), and DHPC-DMPC bicelles (zwitterionic) indicates that binding of verteporfin to C99 is independent the detailed chemistry and structure of the molecules comprising the membrane mimetic. At the same time, interfacial binding of verteporfin to C99 is structurally specific: it is saturable, and affinity can be reduced by mutating specific residues in C99. Moreover, verteporfin was shown not to bind to another single-span membrane protein, the Notch 1 receptor.

Verteporfin was found to inhibit cleavage of C99 by γ-secretase, with an IC_50_ that ranged from 15 μM (near K_D_) to 164 μM, depending on the exact conditions. For γ-secretase, IC_50_—the concentration of compound that inhibits 50% of product formation from an enzyme reaction—will vary with enzyme and substrate concentrations and other assay conditions. In this case, verteporfin was seen to compete with γ-secretase for C99, binding much more effectively in the presence of 1 nM enzyme (IC_50_ 15 ±1 μM) than in the presence of 30 nM enzyme (IC_50_ 164 ± 45 μM).

Under the same conditions where IC50 for inhibition of C99 cleavage was 164 ± 45 μM, verteporfin was seen to stimulate Notch-1 cleavage at concentrations of up to ca. 200 μM, above which verteporfin inhibits cleavage with an IC_50_ value of 240 ± 66 μM, depending on the specific Notch-1 substrate used. In light of our results that verteporfin does not bind directly to Notch-1 and that verteporfin exhibits membrane-disrupting effects at concentrations above 200 μM, we think it is likely that both activation and inhibition of Notch-1 cleavage by different concentrations of verteporfin are the consequence of verteporfin perturbation of the membrane mimetic used in the cleavage assays. This is consistent with previous results showing that the activity of purified γ-secretase is sensitive to membrane conditions, such as phospholipid composition and cholesterol content, as well as to solubilizing detergent type and concentration (67, 68, 69).

Our results suggest the feasibility of finding compounds that bind even more tightly to C99 without binding to Notch-1. This would be a welcome development, as inhibition of Notch-1 receptor cleavage by γ-secretase inhibitors causes severe toxicity that has thwarted efforts to explore these inhibitors as potential Alzheimer’s therapeutics (32, 70, 71, 72).

Verteporfin is a porphyrin-based molecule that is FDA-approved for subfoveal choroidal neovascularization, typically derived from age-related macular degeneration. Commonly sold under the trademark Visudyne, verteporfin is administered intravenously and is absorbed by retinal blood vessels behind the eyes. The patient’s eyes are then irradiated with red light (689 nm), which causes verteporfin to produce radical oxygen species that cause local damage to the blood vessels and enhance their clearance. Beyond its FDA-approved use, verteporfin has several other reported biological effects. For example, when exposed to ambient light, verteporfin can induce cross-linking of proteins in cells, leading to cell death (73). Other effects include but are not limited to inhibition of cellular autophagy (74) and inhibition of cell growth by modulating the Hippo pathway (75, 76). The photochemistry of verteporfin combined with these cytotoxic properties and likely problems with bioavailability dictate that verteporfin cannot be regarded as a promising clinical candidate as an inhibitor of C99 cleavage by γ-secretase, much less as a possible Alzheimer’s therapeutic. Moreover, our results also show that while verteporfin does not directly bind Notch-1, it nevertheless does perturb its cleavage at high concentrations. Despite these obvious flaws in verteporfin as a drug lead or even as a tool compound, our discovery does confirm a previous study (29) that molecules can be found that inhibit γ-secretase cleavage of C99 through direct interaction with this substrate. It can be expected that high throughput screening of much larger compound collections than the library of FDA-approved drugs will lead to the discovery of other C99-specific binders that have more attractive chemical, pharmaceutical, and pharmacological properties. It is hoped that the results of this study will motivate future studies to identify and develop molecules of this class. Such molecules may provide potent tools for sorting out the great complexity of the amyloidogenic pathway, the C99 protein fragment, and its relationship to Alzheimer’s disease (1). Finally, we note our hope that the power of using NMR spectroscopy as a primary tool for detecting molecules that bind to C99 is evident from this work.

## Conclusions

Here we show findings from an NMR-based high-throughput screen that the FDA-approved porphyrin molecule, verteporfin, forms a direct 1:1 complex with the transmembrane domain of the human amyloid precursor protein, C99. It was the only compound out of 1184 molecules that reproducibly bound during titration. The complex is robust and detectable across a variety of membrane conditions and does not bind to the Notch-1 transmembrane domain. Certain mutations in C99 disrupt complex formation. Verteporfin was seen to behave as a selective inhibitor of C99 proteolysis. We think these data further validate the proof of concept that substrate-specific modulators of single-pass transmembrane proteins such as C99 can be discovered by coupling NMR-based high throughput screening to rigorous validation experiments.

## Experimental procedures

### Materials

β-n-Dodecyl melibioside (DDMB; Part# ME12) was purchased from Anatrace. 1-myristoyl-2-hydroxy-sn-glycero-3-phospho-(1′-rac-glycerol) (LMPG; CAT# 858120), 1,2-dimyristoyl-sn-glycero-3-phosphocholine (DMPC; CAT# 850345), and 1,2-dihexanoyl-sn-glycero-3-phosphocholine (DHPC; SKU: 850305) were purchased from Avanti Polar Lipids. Imidazole (CAT# I3386), lysozyme (CAT# L6876), RNAse (CAT# R4875), and DNAse (CAT# DN25) were sourced from Sigma-Aldrich. ^15^NH_4_Cl (CAT# NLM-467-PK) and 99% D_2_O (CAT# DLM-4-PK) were sourced from Cambridge Isotope Labs. Amicon Centrifuge Concentrators 10 kDa molecular weight cutoff (MWCO) spin filters were purchased from Fisher Scientific (CAT# UFC901024). Lauryl betaine (Empigen), 30% solution, was purchased from BOC Sciences (CAT# 66455–29–6). HisPur Ni-NTA resin (CAT# A50591) was purchased from Thermo Fisher. Isopropylthiogalactoside (IPTG: CAT# 367–93–1), dithiothreitol (DTT; CAT# 3482–12–3), and HEPES (CAT# H75030–1000) were purchased from Research Products International. Verteporfin was sourced from SelleckChem (CAT# CL 318952), MedChemExpress (CAT# CL 318952), or Sigma-Aldrich (CAT# SML0534). Tris-HCl (J65594) was sourced from Alpha Aesar. PIPES (P6757), sodium acetate (S2889), ammonium acetate (73,594), potassium phosphate monobasic (P0662), and sodium phosphate dibasic (S9763) were all sourced from Sigma-Aldrich. NMR tubes were sourced from WilMad (WG-3000-4-SJ).

### Buffers

***C99 lysis buffer:*** 25 mM Tris, 150 mM NaCl, pH 7.8

***Notch-1 lysis buffer:*** 40 mM HEPES, 150 mM NaCl, pH 7.8

***Imidazole NMR buffer:*** 25 mM Imidazole, 100 mM NaCl, 0.5 mM EDTA, pH 6.5

***PIPES NMR buffer:*** 50 mM PIPES, 100 mM NaCl, 0.5 mM EDTA, pH 6.5

***Acetate NMR buffer:*** 50 mM NaOAc, 100 mM NaCl, 0.5 mM EDTA, pH 4.5

***GA X-linking buffer:*** 250 mM Imidazole, 100 mM NaCl, pH 7.8

**IM-MS buffer:** 100 mM Ammonium Acetate (AmAc), pH 4.5, 0.5 mM EDTA

### Expression of C-terminally tagged C99

C-terminally hexa-His-tagged C99 with an added tryptophan to increase its light extinction coefficient at 280 nm (I109W C99 was expressed in BL21DE3 strain *E. coli* using a pET21b expression vector system as previously described by Hutchison *et al.* (58). A freshly transformed colony (or a colony from a freshly prepared glycerol stock) was used to inoculate a 250 ml baffled flask containing 80 ml LB starter culture with 50 μg/ml ampicillin. The starter culture was grown at 30 °C overnight in a shaking incubator at 200 rpm for a total of 18 to 20 h. The following morning, 5 ml of the densely grown starter culture was transferred into a 2.8 L baffled flask containing 1 L of ^15^N-NH_4_Cl-labeled minimal media (M9) enriched with 0.4% dextrose, 1xMEM vitamins (from 100× stock [New England Biolabs]), 1 mM MgSO_4_, 0.1 mM CaCl_2_, and 50ug/ml ampicillin. The M9 culture was grown at 37 °C in an incubated shaker at 220 rpm until an optical density (OD) of 0.6–0.8 (usually about 5 h after subculture), at which point IPTG was added to a final concentration of 1 mM. The incubator temperature was then lowered to 20 °C and tumbled overnight. The following morning, the bacteria were harvested by centrifugation at 100,00×*g* for 20 min using a JLA8.1000 rotor in a Beckman J-20 XP centrifuge. The supernatant was discarded, and cell pellets were collected and stored at −80 °C in 50 ml conical vials until further use.

### Purification of C99

C99 pellets were removed from 80 °C and thawed at room temperature for 30 min. The thawed pellet was then resuspended in 10 ml of C99 lysis buffer per gram of wet cell, followed by addition of 1 mM PMSF from a 0.1 M stock in EtOH and 20 μg Lysozyme, 2 μg DNase, and 2 μg RNase from a 500× stock. The sample was then tumbled at 4 °C for 1 h and then sonicated on ice at 60 amps, alternating 5 s on/5 s off, for a total of 10 min. The lysate was then transferred to centrifuge tubes and spun at 50,000*g* for 20 min at 4 °C using a JA25.50 rotor in a Beckman J-20 XP centrifuge. The supernatant was discarded, and the C99-containing pellet was dislodged by spatula into 10 ml of C99 Lysis buffer per gram of pellet. The pellet debris was then fully resuspended using a Dounce homogenizer until the solution was cloudy and white in color. The homogenized solution was again sonicated on ice at 60amps, 5 s on/5 s off, for a total of 10 min, and centrifuged at 500,00*g* for 20 min at 4 °C. The supernatant was discarded, and the pellet (now mostly inclusion bodies) was Dounce homogenized again in 10 ml of C99 Lysis buffer per gram of pellet. The fully resuspended solution was then transferred to 50 ml conical vials, at which point Empigen (lauryl betaine) was added to a final concentration of 3% w/v (from 30% stock). The solution was then tumbled at 4 °C overnight to ensure solubilization of the inclusion bodies.

The following morning, the sample was centrifuged at 500,00*g* at 4 °C for 20 min, keeping the supernatant and discarding the pellet. The supernatant was then tumbled with 1 ml of pre-equilibrated Nickel NTA resin per 50 ml of lysate for 1 h at 4 °C. The resin was then spun down in a table-top centrifuge at 500*g* for 5 min and transferred to a column. The column was first washed with 25 column volumes (CV) of C99 lysis buffer +3% w/v Empigen, then 25 CV of C99 lysis buffer +1.5% w/v Empigen. Once fully drained, the column was then washed/exchanged into 25 CV of C99 lysis buffer +0.05% w/v LMPG (14:0 lyso-myristoylphosphatidylglycerol; Avanti 858120) detergent. Then, weakly bound proteins were removed by adding 25 CV of C99 lysis buffer +0.05% w/v LMPG +10 mM imidazole. The remaining weakly bound proteins were washed away using 25 CV of C99 Lysis buffer +0.05% w/v LMPG +30 mM imidazole, collecting some of the flow-through for an SDS-PAGE sample. ∗Note that LMPG can be exchanged for any desired membrane mimetics before elution, such as DDMB or D6PC/DMPC bicelles. Finally, the protein was eluted from the column using 5–10 CV of C99 lysis buffer +0.2% w/v LMPG +500 mM imidazole. The eluate was then buffer exchanged into NMR Buffer (PIPES or imidazole NMR buffer, depending on the experiment) using an Amicon 10 kDa cutoff centrifugal concentrator. When thoroughly buffer exchanged (after at least three cycles of concentration then 10× dilution), the C-terminally His-tagged I109W C99 concentration was determined by UV_280_ absorbance using the concentrator flow-through as a blank and the 280 nm extinction coefficient (ε) of 11,420 OD units per molar per cm. Typical yields for C99 I109W are approximately 5 mg of pure protein per liter of M9 cell growth. The protein was then concentrated to 200 μM and flash frozen in liquid nitrogen in 250 μl aliquots and stored at −80 °C until further use.

### Expression, purification, and thrombin cleavage C99-NT2 (His tag-cleavable)

Thrombin-cleavable N-terminally His-tagged C99 (C99-NT2) was expressed in BL21DE3 strain *E. coli* using a pET21a expression construct follows the same steps as the C-terminally tagged C99 (inclusion body extraction/purification) mentioned above. C99-NT2 pellets were lysed and purified in the same way as mentioned above for C-terminally His-tagged C99. After elution into C99 lysis buffer +0.2% LMPG +500 mM imidazole, the sample pH was brought up to 8.0 using NaOH, and 500 units of thrombin protease was added to the eluted solution. The sample was tumbled at room temperature overnight to ensure 100% proteolysis, which was confirmed by SDS-PAGE. After cleavage was verified, the imidazole in the solution was buffer exchanged away by at least three cycles of 10× concentration/dilutions into C99 Lysis buffer using a 10kDa cutoff Amicon centrifugal concentrator. After imidazole was diluted to <1 mM, the sample was passed over a fresh NiNTA column equilibrated with C99 lysis buffer, collecting the flow-through. The sample was repassed over the resin at least two more times to ensure that all free His-tag peptides were bound, collecting the flow-through each time. After the final flow-through was collected (which contains the purified untagged C99), the resin was eluted using C99 lysis buffer +500 mM imidazole, collecting the sample in a separate tube. The flow-through sample (containing C99) was then buffer exchanged into NMR buffer (PIPES or imidazole NMR buffer, depending on the experiment) using an Amicon 10 kDa cutoff centrifugal concentrator. When thoroughly buffer exchanged (after at least 3 by 10× dilutions into NMR buffer), the protein concentration was determined by UV_280_ absorbance using the concentrator flow-through as a blank and the UV_280_ nm extinction coefficient (ε) of 5960 units per molar per cm. Typical yields for untagged C99 after thrombin cleavage and repurification were around 2 to 5 mg of pure protein per liter of M9 cell growth. The protein was then concentrated to 200 μM and flash frozen in liquid nitrogen in 250 μl aliquots and stored at −80 °C until further use.

### Expression and purification of Notch-1 TM/JM

His-tagged Notch-1 TM/JM, as previously described by Deatherage *et al.* (44, 77), was expressed in the BL21DE3 Star strain of *E. coli* using a pET21b expression vector system. The expression protocol for this construct follows the same steps as the **C-terminally His-tagged I109W C99** expression protocol mentioned above.

Notch-1 TM/JM pellets were removed from −80 °C and thawed at room temperature for 30 min. The thawed pellet was then resuspended in 10 ml of Notch-1 Lysis buffer per gram of wet cell, followed by the addition of 1 mM PMSF from a 0.1 M stock in EtOH and 20 μg Lysozyme, 2 μg DNase, and 2 μg RNase from a 500× stock. The sample was then tumbled at 4 °C for 1 h, then sonicated on ice at 60 amps, 5 s on/5 s off, for a total of 10 min. DTT (dithiothreitol) and Empigen were then added to the lysate to a final concentration of 1 mM and 3% w/v, respectively. The sample was then tumbled overnight at 4 °C to ensure whole cell solubilization. The next day, the solution was transferred to centrifuge tubes and spun at 500,00*g* for 20 min at 4 °C using a JA25.50 rotor in a Beckman J-20 XP centrifuge.

The following morning, the sample was centrifuged at 500,00*g* at 4 °C for 20 min, keeping the supernatant and discarding the pellet. The supernatant was then tumbled with 1 ml of pre-equilibrated Nickel NTA resin per 50 ml of lysate for 1 h at 4 °C. The resin was then spun down in a table-top centrifuge at 500*g* for 5 min and transferred to a column. The column was first washed with 25 CV of Notch-1 lysis buffer +3% w/v Empigen +1 mM DTT, then 25 CV of Notch-1 Lysis buffer +1.5% w/v Empigen +1 mM DTT. Once fully drained, the column was then washed/exchanged into 25 CV of Notch-1 Lysis buffer +0.05% w/v LMPG (14:0 Lyso PG; Avanti 858120) detergent. Then, weakly bound proteins were removed by adding 25 CV of Notch-1 lysis buffer +0.05% w/v LMPG +25 mM imidazole +1 mM DTT, collecting a sample for an SDS-PAGE sample. The remaining weakly bound proteins were washed away using 25 CV of Notch-1 lysis buffer +0.05% LMPG +65 mM imidazole +1 mM DTT, collecting some of the flow-through for an SDS-PAGE sample. Finally, the protein was eluted from the column using 5–10 CV of Notch-1 lysis buffer +0.2% w/v LMPG +500 mM imidazole +1 mM DTT. The eluate was then buffer exchanged into NMR Buffer (PIPES or imidazole NMR buffer, depending on the experiment) using a 50 ml Amicon 10 kDa cutoff centrifugal concentrator. When thoroughly buffer exchanged (after at least three cycles of 10× concentration/dilutions into NMR buffer), the protein concentration was determined by UV_280_ absorbance using the concentrator flow-through as a blank and the molar extinction coefficient (ε) of 6990. Typical yields for Notch-1 TM/JM are 5 to 10 mg of pure protein per liter of M9 cell growth. The protein was then concentrated to 200 μM and flash frozen in liquid nitrogen in 250 μl aliquots and stored at −80 °C until further use.

#### Screening library of compounds

Compounds were provided by the Vanderbilt University High-Throughput Screening Core using a robotic Echo compound dispensing system. The FDA-approved drug library was acquired from the SelleckChem Company. Solid compounds from SelleckChem were dissolved by the HTS core in DMSO to a final stock concentration of 10 mM, and a 1 μl drop of each compound stock was dispensed per well on 96-well plates. To these predispensed plates, premixed NMR samples were added (see **NMR screening sample preparation**).

#### NMR sample preparation for screening

The preparation of NMR screening samples consisted of final concentrations of 50 μM C99, 2.5% w/v LMPG, 50 mM buffer imidazole, 5% v/v D_2_O, 0.5 mM EDTA, pH 6.5. To ensure that these final conditions were maintained, the individual components of the screening samples were added from stocks in a specific order. For example, to make 10 ml of screening sample in imidazole NMR buffer, the following was added: (1) 10 by 250 μl frozen stocks of 200 μM protein were thawed on ice and transferred to a 15 ml conical vial; (2) 1.25 ml of 20% w/v LMPG stock (dissolved in H_2_O) was added to the conical vial, which was mixed by vortexing; (3) 1 ml of 10× concentrated imidazole NMR buffer was added to the conical vial and mixed by vortexing; (4) the sample was then diluted to 10 ml by adding 4.75 ml of ddH_2_O and 0.5 ml of D_2_O and allowed to reach equilibrium for at least 60 min. This final pool contained 50 μM C99, 25 mM imidazole, 100 mM NaCl, 2.5% LMPG, 5% v/v D_2_O, 0.5 mM EDTA, pH 6.5. At that point, the samples (199 μl for each well) were distributed into 96-well plates containing predispensed compounds, thoroughly mixed by pipette, and the complete 200 μl mixtures were transferred to 3 mm x 4 inch NMR tubes. At this point, each 3 mm NMR tube contained a 200 μl sample of 50 μM C99, 50 μM Compound, 25 mM imidazole, 100 mM NaCl, 2.5% w/v LMPG, 5% v/v D_2_O, 0.5% v/v DMSO, 0.5 mM EDTA, pH 6.5. Loaded NMR tubes were cleaned with 100% Ethanol on a kimwipe, securely capped, and transferred to an NMR tube rack compatible with a Bruker SampleJet system.

### High throughput NMR screening of C99

A Bruker Avance III 600 MHz NMR spectrometer equipped with a cryoprobe (CPTCI) and automated sample changer (SampleJet) was used to collect NMR spectra. Sample details are described above. For high-throughput screening data collection, the ^15^N-^1^H BEST-TROSY (b_trosy3gpph.2) experiment with 64 scans for each of 96 increments and a relaxation delay of 200 ms was run at 318 K for a total data acquisition time of 35 min per sample. To achieve enhanced signal-to-noise ratio, follow-up titrations and control experiments were conducted using the BEST-TROSY pulse program with 200 scans per 96 increments at 318 K, for a total data acquisition time of 110 min per sample. Data were processed using Bruker Topspin 3.6 using a Gaussian window function with a line broadening of -10 and a Gaussian max of 0.1 in both dimensions. NMR data were then further processed and analyzed using NMR-FAM SPARKY, plotted using GraphPad Prism 9, and prepared for publication figures using Affinity Designer.

### NOESY NMR experiments

These data were collected on the more sensitive Bruker Avance 800 MHz and 900 MHz NMR spectrometers both equipped with cryoprobes (CPTCI). A sample was prepared containing 300 μM C99 and 300 μM Verteporfin in 2.5% d^27^-LMPG (deuterated alkyl chains), 50 mM d^4^-Imidazole (deuterated imidazole), 100 mM NaCl, 0.5 mM EDTA, 1 mM DSS, pH 6.5 in 85% D_2_O. The ^1^H-^1^H NOESY experiment was recorded on the 800 MHz spectrometer with a mixing time of 120 ms, relaxation delay of 2 s, 4k × 1k data acquisition matrix, using the standard Bruker pulse program (noesyphpr). Data were processed and analyzed using TopSpin 3.6 and graphically enhanced using Affinity Designer.

### Fitting of NMR-binding data

2D ^15^ N-^1^H HSQC NMR data used to calculate dissociations constant (K_D_) values by first normalizing 2D data using the established formula (78) (Equation 1):

(1) $$\delta \left(1\text{H},15\text{N}\right)=\surd \left(1/2\;\left[\delta_{2}^{H}+.14*\delta_{2}^{N}\right]\right)$$

Where δ(1H,15N) is the square root of linear chemical shifts in each respective dimension, δ_H_ and δ_N_ with the Nitrogen normalization factor of 0.14.

After 2D normalization, the data were fit to a hyperbolic binding isotherm using one of the following formulas depending of the data:

(2) $$Y=\frac{Bmax*X}{\left(K_{D}+X\right)}+NS*X$$

Equation 2 was used for fitting the data shown in Figures 3*E* and S4*C*, where titrations revealed linear shifts at high concentrations of ligand and saturation could not be fully reached. For this equation, Y is the total binding, Bmax is the maximum binding in the same units as Y, X is the added ligand in molar units, K_D_ is the equilibrium dissociation constant in the same units as X, and NS is a linear term to account for either vehicle or nonspecific verteporfin effects.

(3) $$Y=\frac{Bmax*X}{\left(K_{D}+X\right)}$$

Equation 3 was use for fitting the data shown in Figures 2*C* and 3*B*, and 4*C* where chemical shift perturbations saturated during titration and did not have linear shifts due to nonspecific effects. For this equation, Y is the total binding, Bmax is the maximum binding in the same units as Y, X is the added ligand in molar units, K_D_ is the equilibrium dissociation constant in the same units as X. 1D NMR-binding curves were generated by directly inputting 1D chemical shift perturbations directly into the proper version of Equation 2. Peak height data, such as that quantified in Figures 4 and S4, were first generated using the peak-picking function in NMRFAM Sparky, and peak heights as a function of ligand concentration were used to generate a K_D_ by addition into Equation 2.

### Glutaraldehyde (GA) cross-linking

C-terminally His-tagged C99 was expressed from *E. coli* as mentioned above, and the sample was eluted into a buffer containing 0.1% w/v LMPG, 350 mM Imidazole, 100 mM NaCl, pH 7.8. The protein sample was buffer swapped into GA X-linking buffer containing 0.25% w/v LMPG, 250 mM imidazole, 100 mM NaCl, pH 7.8, and the protein was concentrated to a stock of 300 μM C99. Protein stocks were either used right away or flash frozen in LN_2_ and stored at −80 °C until further use.

On the day of the experiment, fresh glutaraldehyde (from 25% w/v ampoule) was used to prepare serial dilutions and stored on ice. Reaction volumes were set at 50 μl, containing 30 μM C99, 25 mM Imidazole, 100 mM NaCl, pH 7.8, and 2.5% w/v LMPG. For a typical GA reaction setup, the following components were added to Eppendorf tubes in this specific order: 5 μl of 300 μM C99 was added to the bottom of the tube, followed by 6 μl of 20% w/w LMPG solution, then the sample was diluted to 50 μl using 39 μl of GA X-linking buffer. The sample was mixed by vortex and allowed to sit for at least 30 min. After incubation, 1 μl of Verteporfin stock (typically 10 mM in DMSO) was added to the mixture, vortexed, and allowed to incubate for at least another 30 min in the dark. Finally, 1 μl GA was added from the corresponding stock (depending on the concentration needed) to the side of the Eppendorf tube. After all the samples had GA droplets added to the sides of the corresponding tubes, the drops were plunged to the bottom by centrifugation at 10,000*g* for 10 s, and the tubes were mixed by vortex and allowed to react for 30 min. After the reaction time was completed, the reactions were quenched using 2 μl of 1 M Tris/Glycine buffer and allowed to sit for at least 5 more minutes to ensure complete quenching. Then, SDS loading buffer was added directly to the reaction tubes, and the samples were visualized using SDS-PAGE and analyzed by densitometry using ImageJ. Data were fit using the following one-phase exponential decay function (Equation 4):

(4) $$Y=\left(Y_{0}-Plateau\right)*e^{-K*X}+Plateau$$

Where Y starts at Y_0_ and decays down to the Plateau (Y_final_), X is concentration, and K is the rate constant in the reciprocal units of X.

### Ion mobility–mass spectrometry (native mass spectrometry)

IM-MS data were collected using a Synapt G2 HDMS IM-Q-ToF mass spectrometer (Waters), with a direct infusion nESI source set to positive ion mode. The instrument settings were tuned to generate intact C99–verteporfin complex ions while completely dissociating them from detergent LMPG micelles, including appropriately tuned settings for the sampling cone 80 V, trap cell accelerating potential 80 V, and the transfer cell accelerating potential 70 V. The trapping cell wave velocity and height were 116 m/s and 0.1 V, respectively. The IMS wave velocity and height were 250 m/s and 15 V, respectively. The transfer cell wave velocity and height were 300 m/s and 10 V. Prior to analysis, C99 in LMPG micelles at a protein concentration of 20 μM C99 and 100X CMC LMPG or DDMB samples were prepared by incubating 20 μM C99 with 100X CMC LMPG for 30 min, then titrating in DMSO or Verteporfin and incubating in the dark overnight. Verteporfin-binding buffer conditions were 200 mM Ammonium Acetate at pH 4.5. For all C99–verteporfin LMPG micelles measured at 0 μM, 10 μM, 20 μM, 30 μM, 40 μM, 50 μM, and DMSO verteporfin concentrations, C99 was observed as monomeric and dimeric species, with the monomeric species having a charge state distribution between 9+ - 4+ and the dimeric species having a charge state distribution between 12+ - 8+.These charge state distributions were observed to persist across all verteporfin and DMSO concentrations and were extracted into a text-based format using TWIM extract to compute the relative intensity of each species at a given weight percentage.

### γ-Secretase assay

The γ-secretase-dependent proteolysis assays with both substrates (C100-FLAG and HexaHis Notch 1 JM/TM) were performed in a weak zwitterionic detergent (CHAPSO, 3-[(3- cholamidopropyl)dimethylammonio]-2-hydroxy-1-propanesulfonate) solubilized conditions (79, 80). C100 is C99 with an added N-terminal Met residue. Stock solution of purified γ-secretase (81, 82) was diluted to 30 nM in standard assay buffer containing 50 mM HEPES (pH 7.0), 150 mM NaCl, 0.1% DOPC (phosphatidylcholine), 0.025% DOPE (phosphatidylethanolamine), and 0.25% CHAPSO) and incubated at 37 ^°^C for 30 min, whereupon stock solution of verteporfin or inhibitor (LY411,575) was added to achieve the desired concentration with 1% final DMSO concentration. Proteolytic reactions were initiated by adding purified substrates (either C100-FLAG(79) or HexaHis-Notch 1 JM/TM(77)) and incubating at 37 °C (16 h for C100Flag and 4 h for HexaHis-Notch 1 JM/TM). Reactions were quenched with SDS, and the cleaved intracellular domain products (AICD-FLAG released from C100-FLAG or truncated intracellular domain (NICD) released from the proteolysis of Hexa-His Notch 1 JM/TM) were visualized by Western blot using specific primary antibodies, anti-FLAG antibody for FLAG-tagged AICD, and neoepitope-specific anti-V1744 antibody for NICD (80, 83, 84).

### IC_50_ measurements

Purified γ-secretase was diluted in standard assay buffer to specified concentrations and incubated at 37 °C for 30 min, followed by the addition of stock solution of verteporfin in DMSO to achieve various final verteporfin concentrations and 2% final DMSO concentration. Purified C99 FLAG was then added to start the proteolytic reactions, which were incubated for 2 h at 37 °C. The concentrations of Aβ40 produced during the proteolysis of C100 FLAG by γ-secretase were determined using a specific sandwich ELISA (Invitrogen). Aβ40 concentrations were plotted as a function of verteporfin concentration, and the resultant sigmoidal curves were fitted to Equation 5 to determine IC_50_ values (80, 83).

(5) $$\;Y=Min+\frac{\left(Max-Min\right)}{1+{\left(\frac{X}{IC50}\right)}^{Hill\;Coefficient}}$$

### Dynamic light scattering

Samples were made according to NMR sample conditions. Solutions were first filtered with a 0.2 μm syringe filter then centrifuged at 100,00*g* to remove insoluble debris. The solution was then added to the inner chamber of the disposable DLS cuvette. The disposable DLS cuvette was capped and loaded in the DLS instrument. Experiments were carried out at 25 °C. The DLS software (Dynamics 7.5 software; Wyatt Technologies) calculates an apparent hydrodynamic radius (*R*_*h*_) *via* the Stokes–Einstein equation (Equation 6):

(6) $$R_{h}=\frac{kT}{6\pi \eta D_{t}}$$

where k is Boltzmann’s constant, T is the absolute temperature, η is sample viscosity, and D_t_ is the diffusion coefficient. The apparent molecular weight of the detergent or verteporfin particles was calculated in the Dynamics software, as based on the measured apparent radius of hydration, using the assumption that the density of the particle was similar to that of a globular protein.

### Preparation of protein structures for docking

The full-length structure of C99 in micelles was obtained from the RCSB PDB Database (PDB: 2LP1). The PDB file was relaxed within an implicit membrane in Rosetta 3.13 using RosettaMP protocols (85, 86, 87). Implicit membrane position and thickness were set to span residues A701-V721. Five hundred relaxed models were generated using the FastRelax mover within the implicit membrane and scored by an all-atom membrane score function. The model with best total score was selected as the representative to be used for docking simulations.

### Preparation of verteporfin conformers for docking

The 2D structure of verteporfin was obtained from PubChem (CID: 11980904) and converted to a 3D conformer using CORINA. BCL Conformer Generator was then used to create a conformer library (250 conformers) from this 3D template (88).

### Local docking of verteporfin to C99

The verteporfin conformer library was docked to the relaxed C99 model within an implicit membrane in Rosetta 3.13 by coupling RosettaLigandDocking and RosettaMP protocols (85, 86, 87, 89, 90, 91). Conformers were initially placed between the C99 transmembrane helix and the C-helix to be near perturbed residues identified by ^15^N-^1^H TROSY experiments. Large conformer moves were sampled by a 1000-step low resolution Monte Carlo search of the binding pocket using the Transform mover. Next, six cycles of the HighResDocker Mover were used to sample alternate rotamers near the ligand and perform small ligand sampling, repacking the protein–ligand interface every third cycle. Finally, the FinalMinimizer Mover was used to perform in-membrane gradient-based minimization of the pose, refining it to a final output model. In total, 65,000 models were generated in this manner and scored within a membrane all-atom score function as during relaxation.

#### Model evaluation and RMSD analysis

Docked models were sorted by their within-membrane interface scores, and the best-scoring model was selected as the representative structure. All models were then evaluated against the best scoring model by plotting their interface scores *versus* their RMSD to the best-scoring model. Plots were generated using the ggplot2 package (version 3.3.2) in R (version 4.0.2).

## Data availability

All main text data are in this manuscript. Supplemental data and information are in the corresponding Supplemental Information document. Correspondence and requests for materials should be addressed to the corresponding author (chuck.sanders@vanderbilt.edu).

## Supporting information

This article contains supporting information (Figs. S1–S7).

## Conflict of interest

The authors declare that they have no conflicts of interest with the contents of this article.
