# Supplementary material for: Verteporfin is a substrate-selective γ-secretase inhibitor that binds the amyloid precursor protein transmembrane domain
Source: J Biol Chem. 2022 Mar 3;298(4):101792. doi: 10.1016/j.jbc.2022.101792 (PMC8968665; doi:10.1016/j.jbc.2022.101792)
Supplement: Supplemental Figures S1–S7 [file mmc1.pdf]

Supporting Information for:

Verteporfin Binds the Amyloid Precursor Protein Transmembrane Domain and Is a Substrate-Selective γ-Secretase Inhibitor

Manuel A. Castro, Kristine F. Parson, Ilyas Beg, Mason C. Wilkinson,
Kamila Nurmakova, Iliana Levesque, Markus W. Voehler,
Michael S. Wolfe, Brandon T. Ruotolo, Charles R. Sanders*

***To whom correspondence should be addressed:**
chuck.sanders@vanderbilt.edu

This document includes:

Supporting Figures S1-S7

Figure S1.

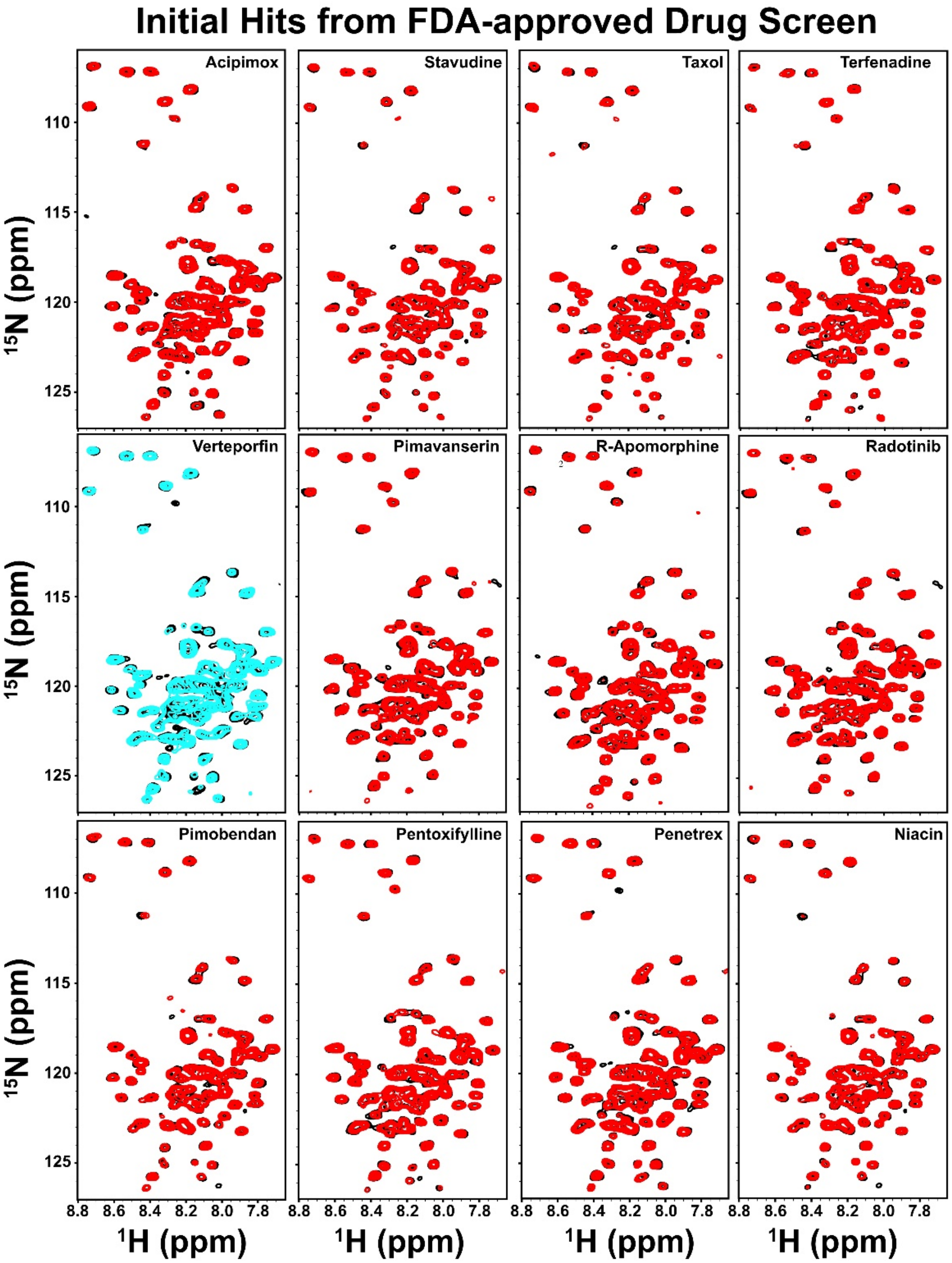

Figure S1. TROSY NMR spectral overlays of representative initial hit spectra from primary screen. Spectra for hit compound-containing samples are shown in red, overlaid onto the vehicle control (DMSO) spectrum in black. Initial hits were recognized based on chemical shift perturbations at one or several C99 residues. The initial NMR screen of approximately 1,200 compounds produced 20 hits that were followed-up on (12 shown here). Verteporfin (cyan spectrum) was seen to induce spectral perturbations such as peak broadening and shifting to degrees that were more obvious and widespread than induced by any of the other hits.

Figure S2.

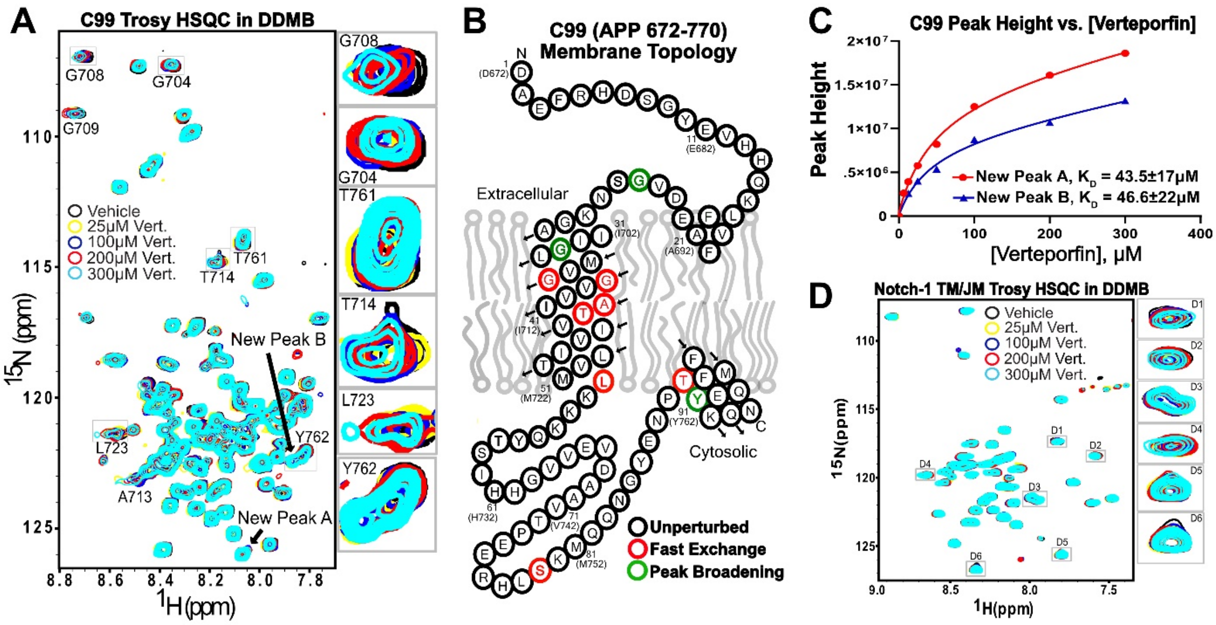

Figure S2. Verteporfin binds untagged C99 in DDMB micelles with a K_D of 45 ± 22 µM and does not bind the Notch-1 TM/JM domain. A) Five overlaid C99 TROSY NMR spectra at varying concentrations of verteporfin. **B**) C99 topology diagram highlighting residues that undergo perturbations in response to addition of verteporfin. **C**) Chemical shift perturbations from the verteporfin titration shown in panel A. **D**) Notch-1 TM/JM TROSY spectral overlay at varying concentrations of verteporfin. Insets D1-D6 highlight minor spectra changes oberserved in Notch-1 spectra at high concentrations of verteporfin, likely arising from non-specific membrane perturbations. These data are n=1, where the uncertainties for the K_D in C are the error of the fit.

Figure S3.

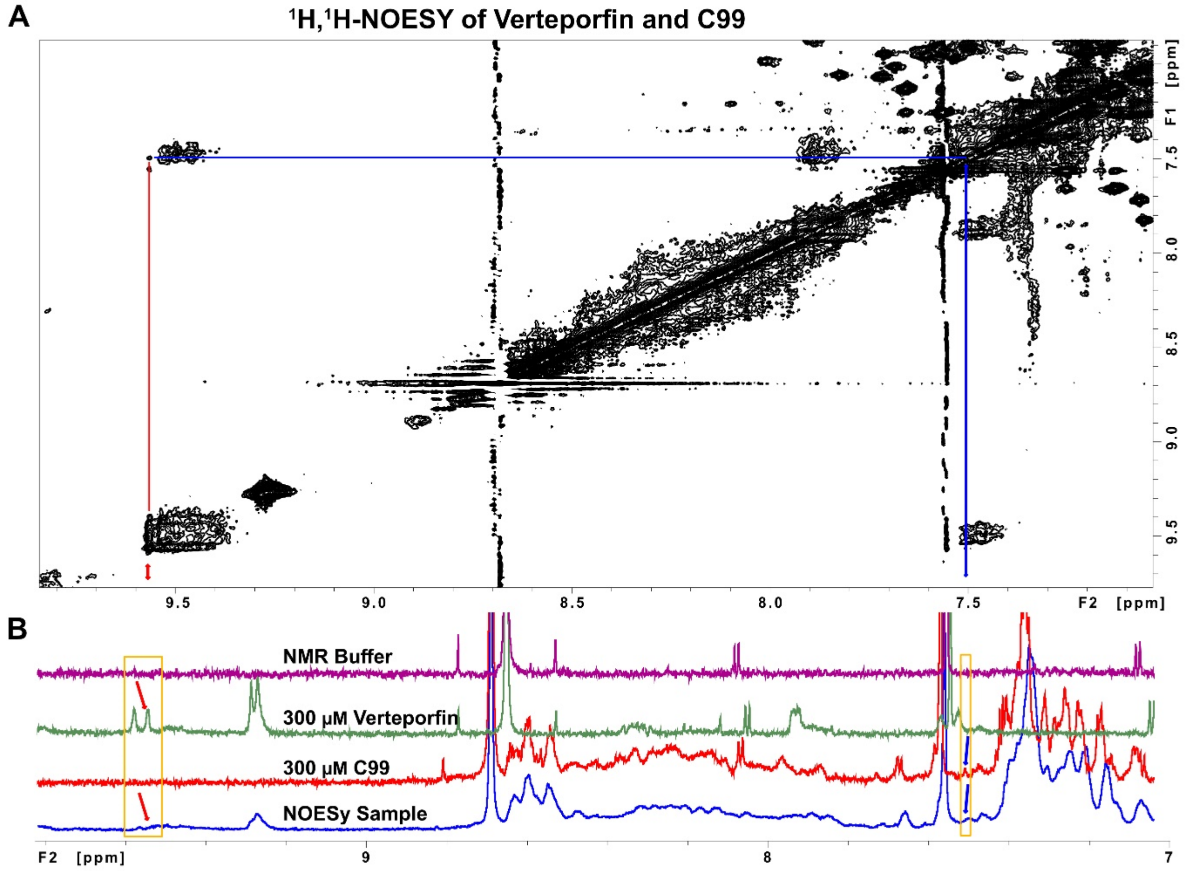

Figure S3. ^{1}H,^{1}H-NOESY of the C99-verteporfin complex. A) 2D ^{1}H,^{1}H-NOESY experiment applied to a sample containing 300µM C99 and 300 µM verteporfin revealed cross-peaks between well-resolved aromatic verteporfin and C99 resonances. **B**) ^{1}H spectra of four samples containing either NMR buffer only (purple spectrum), 300 µM verteporfin in NMR buffer (green spectrum), 300 µM C99 in NMR buffer (red spectrum), and the NOESY sample containing both verteporfin and C99 (blue). The verteporfin inner-ring proton resonance (green spectrum, orange box, 9.55 ppm) exhibits an NOE interaction with a C99 amide resonance (red spectrum, orange box, 7.5 ppm). The corresponding resonances in the NOESY sample (blue spectrum) are broadened due to intermediate exchange, similar to what was seen for some C99 residues in the corresponding TROSY NMR spectra. These data were only collected once.

Figure S4.

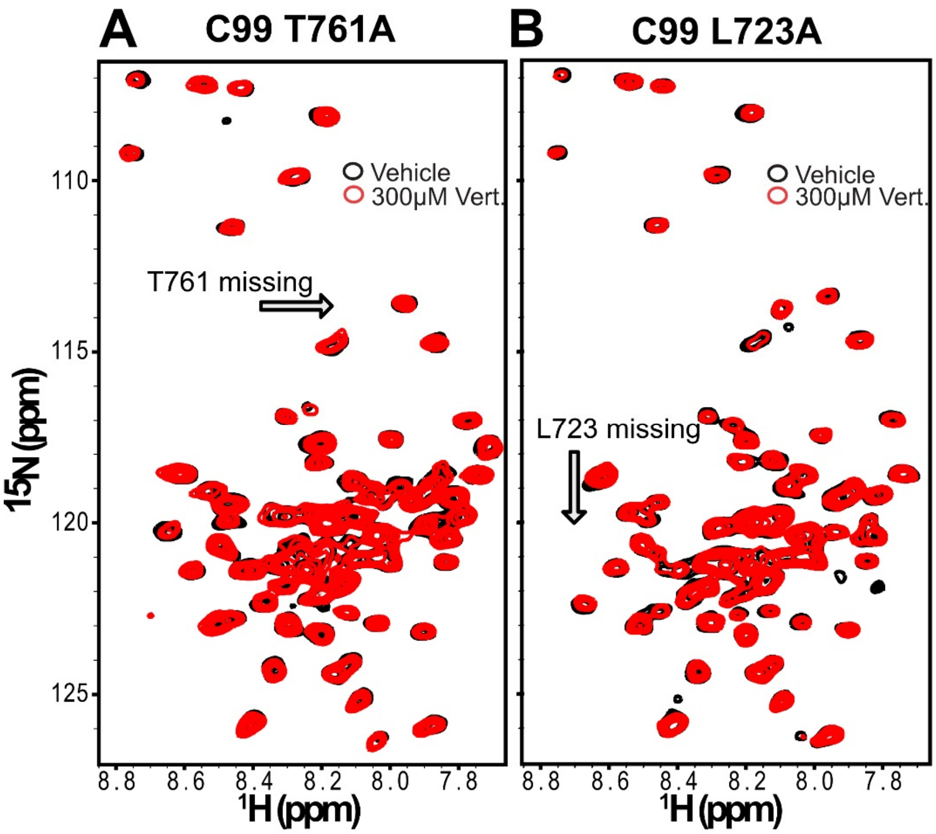

Figure S4. C99 point mutations that ablate affinity for verteporfin. A) Two NMR spectra of C99 T761A with vehicle-treated in black overlaid by spectra for samples containing 300 μM verteporfin in red. There were no chemical shift perturbations seen at any concentrations for this mutant, suggesting complete lack of binding. **B**) Two NMR spectra of C99 L723A, with vehicle-treated in black overlaid by the red spectrum representing the 300 μM verteporfin condition. There were only noisy shifts seen for this construct when treated with verteporfin, suggesting a nearly complete lack of binding. These data are representative spectra taken from the highest and lowest (zero) titration point from the series. The titrations were done once (n=1) per mutant.

Figure S5

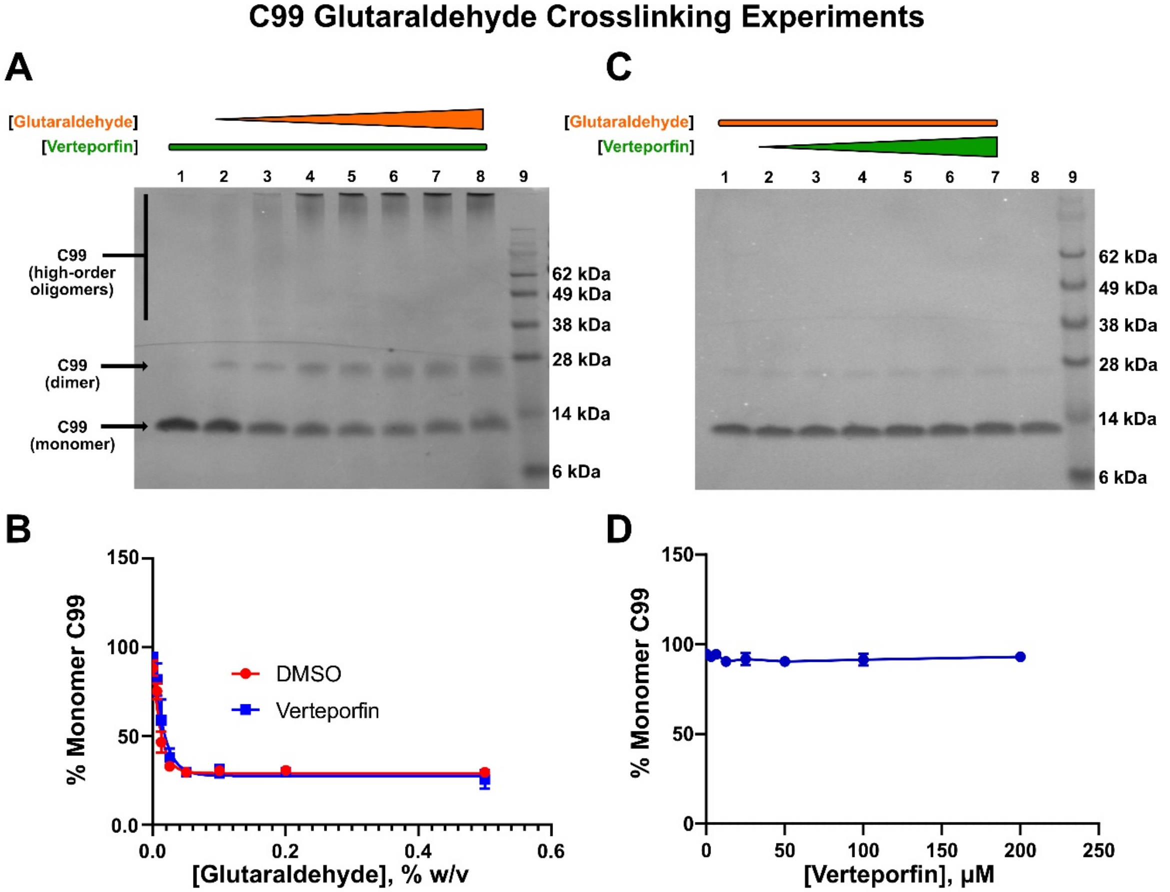

Figure S5. **Glutaraldehyde crosslinking of C99 reveals no change in oligomeric state in response to verteporfin. A**) SDS-PAGE gel of crosslinking experiment at constant 100 µM verteporfin and treatment with increasing levels of glutaraldehyde from 0-0.5% w/v at final dilution. **B**) Quantified C99 band intensities from gel shown in A, indicating no changes of the oligomeric state of C99 in response to verteporfin treatment when compared to the DMSO treated control. **C**) SDS-PAGE gel of crosslinking experiment with C99 being treated with increasing amounts of verteporfin at a constant low concentration (0.0064% w/v) of glutaraldehyde, where basal levels of dimer crosslinking can be seen. **D**) Quantification of the gel from B also verifies that C99 remains a robust monomer in response to titration with verteporfin. All data shown here are representative of n=3 technical replicate experiments, which were quantified by densitometry using ImageJ, where the error bars denote the standard deviation of the replicate values.

Figure S6

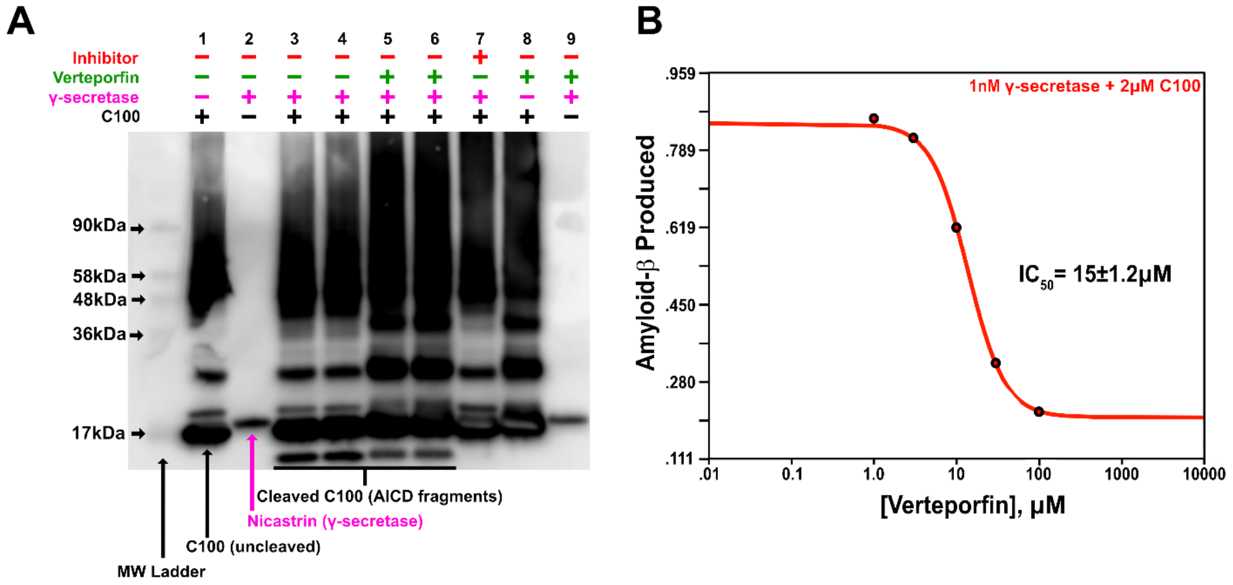

Figure S6. Verteporfin inhibits γ-secretase cleavage of C100-Flag (C99) in low enzyme and substrate conditions. C100-Flag is recombinant C99 with an N-terminal start methionine and a C-terminal Flag epitope tag. **A**) Anti-Flag tag Western blot of 2 μM C100-Flag cleavage by 1 nM purified γ-secretase. Cleavage of C100-Flag proceeds as expected in lanes 3 and 4 (bottom band; AICD-Flag = intracellular domain product of C100-Flag cleavage by γ-secretase). However, upon addition of 100 μM verteporfin, the cleavage efficiency is greatly reduced (lanes 5 and 6), but not as fully as seen in lane 7, which contained 30 nM of the potent γ-secretase-inhibitor, LY411,575. **B**) Sandwich ELISA of Aβ40 production from C100-Flag cleavage by purified γ-secretase performed as in panel A. Inhibition by verteporfin is concentration-dependent. Curve fitting reveals a half-maximal inhibitory constant (IC_{50}) of 15 ± 1 μM verteporfin. These data are from single n=1 experiment, and the error was interpolated from the error of the fit.

Figure S7.

A

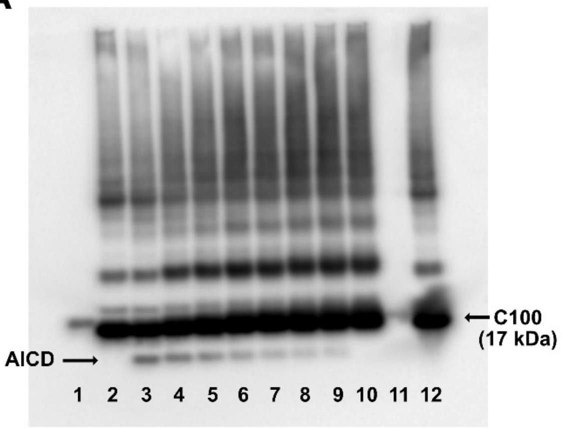

B

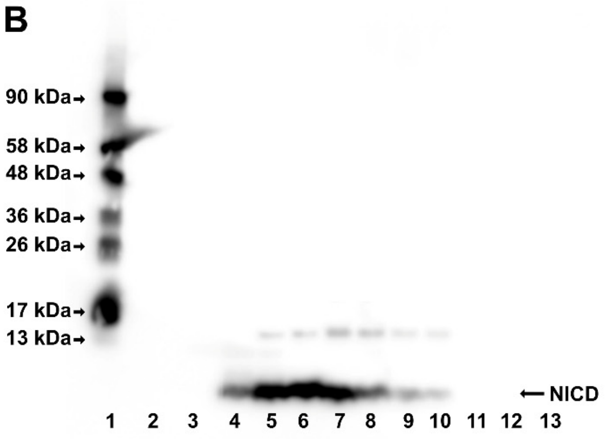

1) 30nM γ-secretase
2) 5μM C100
3) 5μM C100 + 30nM γ-secretase
4) 5μM C100 + 30nM γ-secretase + 50μM Verteporfin
5) 5μM C100 + 30nM γ-secretase + 100μM Verteporfin
6) 5μM C100 + 30nM γ-secretase + 200μM Verteporfin
7) 5μM C100 + 30nM γ-secretase + 300μM Verteporfin
8) 5μM C100 + 30nM γ-secretase + 500μM Verteporfin
9) 5μM C100 + 30nM γ-secretase + 1000μM Verteporfin
10) 5μM C100 + 1000μM Verteporfin (No Enzyme)
11) 30nM γ-secretase + 1000μM Verteporfin (No Substrate)
12) 5μM C100 + 30nM γ-secretase
+ Inhibitor (30nM Ly411,575)

1) Ladder
2) 30nM γ-secretase
3) 20μM Notch-1
4) 20μM Notch-1 + 30nM γ-secretase
5) 20μM Notch-1 + 30nM γ-secretase + 50μM Verteporfin
6) 20μM Notch-1 + 30nM γ-secretase + 100μM Verteporfin
7) 20μM Notch-1 + 30nM γ-secretase + 200μM Verteporfin
8) 20μM Notch-1 + 30nM γ-secretase + 300μM Verteporfin
9) 20μM Notch-1 + 30nM γ-secretase + 500μM Verteporfin
10) 20μM Notch-1 + 30nM γ-secretase + 1000μM Verteporfin
11) 20μM Notch-1 + 1000μM Verteporfin (No Enzyme)
12) 30nM γ-secretase + 1000μM Verteporfin (No Substrate)
13) 20μM Notch-1 + 30nM γ-secretase
+ Inhibitor (30nM Ly411,575)

Figure S7. Uncropped γ-secretase assay blots from main text Figure 6. A) Entire image of C100 γ-secretase assay blot. Legend below annotates the components of each well. Amyloid intracellular domain (AICD) bands can be seen at the bottom in lanes 3-9, where enzyme and substrate are present. **B**) Entire image of Notch-1 TM/JM γ-secretase assay blot. Notch intracellular domain (NICD) bands are seen in lanes 4-10, where substrate and enzyme are present.
